# Dimer Asymmetry and Light Activation Mechanism in *Brucella* Blue-Light Sensor Histidine Kinase

**DOI:** 10.1128/mBio.00264-21

**Published:** 2021-04-20

**Authors:** Jimena Rinaldi, Ignacio Fernández, Heewhan Shin, Gabriela Sycz, Semini Gunawardana, Indika Kumarapperuma, Juan M. Paz, Lisandro H. Otero, María L. Cerutti, Ángeles Zorreguieta, Zhong Ren, Sebastián Klinke, Xiaojing Yang, Fernando A. Goldbaum

**Affiliations:** a Fundación Instituto Leloir, IIBBA-CONICET, Buenos Aires, Argentina; b Department of Chemistry, University of Illinois at Chicago, Chicago, Illinois, USA; c Plataforma Argentina de Biología Estructural y Metabolómica PLABEM, Buenos Aires, Argentina; d Department of Ophthalmology and Vision Sciences, University of Illinois at Chicago, Chicago, Illinois, USA; Massachusetts Institute of Technology

**Keywords:** photoreceptor, sensory histidine kinase, crystallography, dimer asymmetry, light activation mechanism

## Abstract

The ability to sense and respond to environmental cues is essential for adaptation and survival in living organisms. In bacteria, this process is accomplished by multidomain sensor histidine kinases that undergo autophosphorylation in response to specific stimuli, thereby triggering downstream signaling cascades. However, the molecular mechanism of allosteric activation is not fully understood in these important sensor proteins. Here, we report the full-length crystal structure of a blue light photoreceptor LOV histidine kinase (LOV-HK) involved in light-dependent virulence modulation in the pathogenic bacterium Brucella abortus. Joint analyses of dark and light structures determined in different signaling states have shown that LOV-HK transitions from a symmetric dark structure to a highly asymmetric light state. The initial local and subtle structural signal originated in the chromophore-binding LOV domain alters the dimer asymmetry via a coiled-coil rotary switch and helical bending in the helical spine. These amplified structural changes result in enhanced conformational flexibility and large-scale rearrangements that facilitate the phosphoryl transfer reaction in the HK domain.

## Abstract (precis)

Bacteria employ two-component systems (TCSs) to sense and respond to changes in their surroundings. At the core of the TCS signaling pathway is the multidomain sensor histidine kinase, where the enzymatic activity of its output domain is allosterically controlled by the input signal perceived by the sensor domain.

## INTRODUCTION

Widespread in bacterial signaling, two-component systems (TCSs) perceive and transduce various chemical and physical stimuli to trigger appropriate cellular responses (1, 2). TCSs typically consist of a sensor histidine kinase (SHK) and a cognate response regulator (RR). SHKs undergo autophosphorylation in response to an input signal such as light, a small-molecule ligand, or a mechanical force, whereas RRs activate the downstream responses upon receiving a phosphoryl group from the SHK. Some SHKs also have phosphatase activity that removes the phosphoryl group from the RR (2), allowing the TCS to restore. SHKs are often multidomain signaling proteins organized in a modular architecture that undergo allosteric transition between two signaling states: (i) the active or ON state, with autophosphorylation and phosphotransferase activities, and (ii) the inactive or OFF state, with phosphatase activity (1, 2). Most SHKs are homodimeric, in which the sensor domains, linker helices, and histidine kinase (HK) domains are juxtaposed along a central helical spine at the dimer interface. The HK domain consists of two subdomains called dimerization and histidine phosphotransfer (DHp) and catalytic and ATP binding (CA). Despite the vast amount of structural and biochemical studies available in the literature, many important questions regarding the allosteric activation of SHKs remain unanswered. Specifically, how the structural signals propagate or amplify within a full-length dimeric framework is not fully understood at the molecular level. Currently, the atomic resolution information on SHK structures consisting of both sensor and HK domains is rather limited. Thus, the detailed depiction of structural changes between different signaling states in the same SHK system is critical for elucidating the molecular mechanisms of SHK activation.

**FIG 1:**
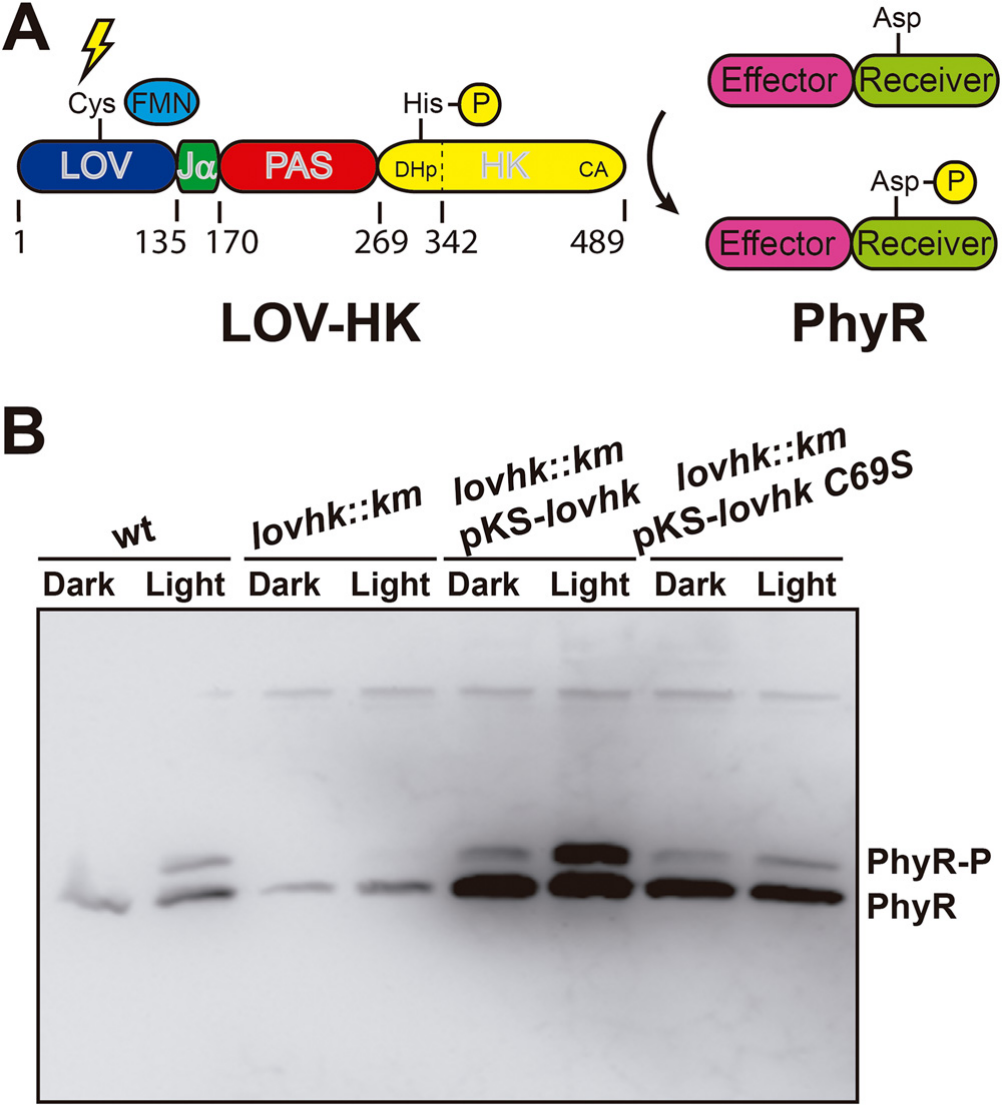
*In vivo* light-gated activation of the two-component system. (A) Schematic representation. The domain architecture of the LOV-HK photoreceptor and the PhyR response regulator is shown, together with the numbering of domain and subdomain boundaries of LOV-HK. (B) Intracellular PhyR∼P levels. The B. abortus 2308 wild-type (wt), the *lovhk* mutant (*lovhk*::*km*), the *lovhk* mutant complemented with the pKS-*lovhk* plasmid (*lovhk*::*km* pKS-*lovhk*), and the *lovhk* mutant complemented with the pKS-*lovhk* C69S (*lovhk*::*km* pKS-*lovhk* C69S) were grown in dark and light conditions and analyzed by Phos-tag gel electrophoresis and Western blotting with an anti-PhyR antibody. The gel corresponds to one representative experiment of two independent assays.

We employ light-oxygen-voltage histidine kinase (LOV-HK) as a model system to investigate the structural changes in SHKs in response to an input signal. LOV-HK is a blue light photoreceptor from Brucella abortus, a pathogenic α-2-proteobacterium that can be transmitted from cattle to humans causing brucellosis. The light dependent virulence enhancement in B. abortus has been linked to upregulation of the LOV-HK activity (3, 4). LOV-HK is thought to play an important role in modulating the bacterium-host interactions in both plants and animals (5–12) via the response regulator PhyR, a key element of the general stress response in proteobacteria (13–17). B. abortus LOV-HK comprises an N-terminal blue light-sensing LOV domain, a central PAS (Per-Arnt-Sim) domain of unknown function and a C-terminal HK domain (Fig. 1A). The LOV and PAS domains are connected through a long linker helix denoted the Jα helix, while the HK domain belongs to the HWE family. SHKs in the HWE family are widespread in alphaproteobacteria, although they only represent about 3% of the HK superfamily (2). Upon blue light illumination, B. abortus LOV-HK photobleaches as a result of the adduct formation between its flavin mononucleotide (FMN) chromophore and an adjacent conserved cysteine residue as in other LOV proteins (18, 19). This local light-induced conformational change (20–25) then propagates to alter the enzymatic activities of the C-terminal HK domain in an allosteric manner. Conformational changes within the HK domain based on the comparison between inactive and active (Michaelis complex) structures have been proposed for B. abortus LOV-HK (26, 27) and other systems (28, 29), where the repositioning of the CA subdomain is entailed. The autophosphorylation in SHKs can either occur intermolecularly (in *trans*) or intramolecularly (in *cis*), with the latter case occurring in B. abortus LOV-HK (27). Compared to most SHKs involved in transmembrane signaling, B. abortus LOV-HK is soluble and can be activated by light; thus, it is well suited for mechanistic dissection of signal perception, transduction, and allosteric activation of SHKs by biophysical methods.

Here, we present extensive structural studies on B. abortus LOV-HK in both full-length and truncated contexts. We have determined the crystal structure of the full-length protein in the light state. We have also captured the light-induced structural changes in the truncated LOV-PAS construct via a joint analysis of 22 crystallographic data sets collected from light-sensitive crystals under dark and light conditions. Direct comparisons between the dark and light structures allowed us to dissect how light detection in the N-terminal LOV domain is coupled to the autophosphorylation of the C-terminal HK domain. Our findings demonstrate that the light activation of LOV-HK is accompanied by a series of structural events in which subtle light-induced structural signals that originated at the sensor LOV domains are amplified via the parallel dimeric framework, resulting in a significant increase in dimer asymmetry as the LOV-HK photoreceptor transitions from a dark slightly asymmetric inactive state to a highly asymmetric light state.

## RESULTS

### *In vivo* function of LOV-HK.

The increased virulence of B. abortus under light has been attributed to LOV-HK (4). We have demonstrated *in vitro* that LOV-HK binds and phosphorylates the response regulator PhyR (17) (Fig. 1A). To establish the *in vivo* function of this light-gated TCS, we examined how the PhyR phosphorylation is affected by light in B. abortus. We found in Western blots with anti-PhyR antibodies (17) that the wild-type B. abortus strain displayed higher intracellular levels of phosphorylated PhyR under light growth conditions than in the dark (Fig. 1B). In the knockout *lovhk* strain, the total amount of PhyR was reduced, and no phosphorylated PhyR was detected under either dark or light conditions. When the knockout strain was complemented with either wild-type LOV-HK or LOV-HK-C69S (a “blind” variant in which the conserved Cys69 residue is replaced by a serine), the PhyR expression was significantly increased, and the phosphorylated PhyR bands reappeared. Not surprisingly, the upregulation of the PhyR phosphorylation by light was only observed in the strain complemented with wild-type LOV-HK and not in the strain complemented with LOV-HK carrying the C69S mutation. These observations are consistent with our earlier finding that LOV-HK positively regulates the expression of PhyR (17). Taken together, the regulation of the intracellular levels of phosphorylated PhyR by LOV-HK supports that the light-dependent virulence in B. abortus is mediated by the TCS pathway consisting of LOV-HK and its cognate signaling partner PhyR *in vivo*.

### The N-terminal helix from the LOV domain promotes formation of the parallel dimer.

**FIG 2:**
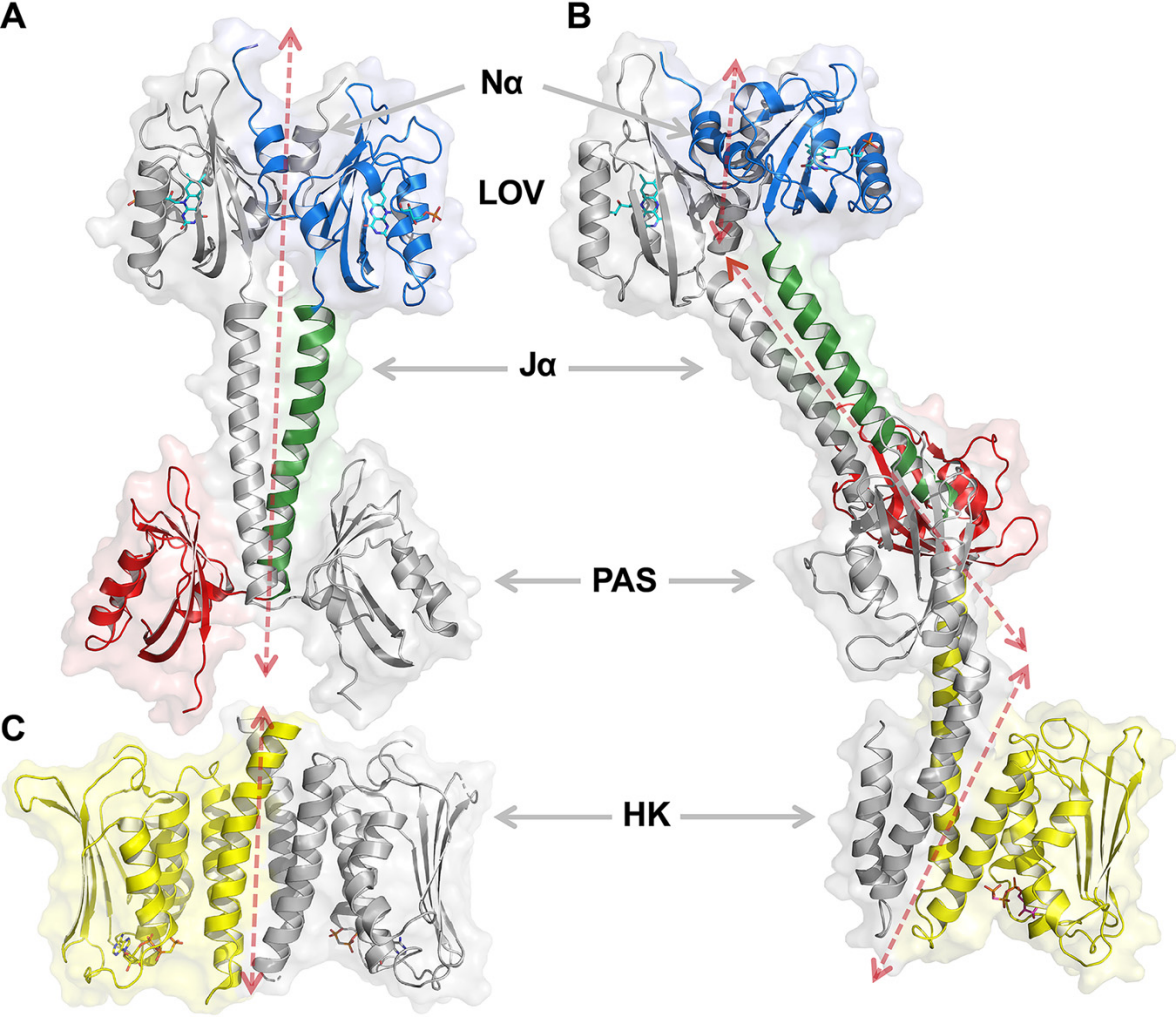
Crystal structure of LOV-PAS and LOV-PAS-HK. (A) Ribbon diagram of dark-adapted LOV-PAS shows a parallel dimer structure in which the juxtaposed LOV and PAS domains are tethered via two long Jα helices with subtle dimer asymmetry. One subunit is colored according to the domain architecture, while the other is rendered in gray. Ligands are depicted in sticks (see the main text for details). The dashed arrow sketches the trace of the helical spine. (B) Ribbon diagram of light LOV-PAS-HK shows a highly asymmetric dimer with a significant distortion in the helical spine that extends into the HK domain. (C) Ribbon diagram of the isolated HK domain in the inactive state, as published previously (PDB code 5EPV, chains A and B). The location and orientation of this panel give rise, in visual combination with panel A, to an estimate of the structure of the full-length protein in the dark.

The blue light sensing LOV domain is flanked by α helices at both its N and C termini, denoted the Nα and Jα helices, respectively (Fig. 2; see also Fig. S1 in the supplemental material). To evaluate the role of these flanking regions of the LOV domain in B. abortus LOV-HK, we designed different constructs corresponding to the LOV core harboring different N- and C-terminal extensions. We crystallized and obtained the structure of a “blind” construct consisting of the Nα helix, half of the Jα helix, and the LOV core (LOVN13J21 C69S, Table 1). The structure reveals a parallel dimer, in which the Nα helices intertwine and interact with the β sheet of the core (see Fig. S1A). Such dimeric association is similar to those observed in other LOV and PAS domains (8, 30–34), but it contrasts with the isolated core of the LOV domain, which forms an antiparallel and unstable dimer (25).

**TABLE 1:** X-ray diffraction data collection and refinement statistics

Parameter	LOV-N13J21 C69S	LOV-PAS (dark)	LOV-PAS (light)	LOV-PAS-HK (light)
Data collection
Synchrotron source	SOLEIL	SOLEIL	APS	SOLEIL
Beamline	PROXIMA-1	PROXIMA-2A	21-ID-G	PROXIMA-2A
Wavelength (Å)	0.9786	0.9801	0.9787	0.9801
Temp (K)	100	100	100	100
Detector	PILATUS 6M	EIGER X 9M	MARMOSAIC 300	EIGER X 9M
Crystal-detector distance (mm)	463.80	216.01	350.00	317.67
Rotation range/image (°)	0.2	0.1	1.0	0.1
No. of frames	1,000	3,600	240	4,000
Exposure time/image (s)	0.200	0.025	3	0.025
				
Indexing and scaling
Cell parameters
*a* (Å)	66.27	108.42	109.97	95.96
*b* (Å)	95.86	56.93	56.81	104.66
*c* (Å)	107.59	114.60	115.91	164.83
α (°)	90	90	90	90
β (°)	90	103.36	103.32	90
γ (°)	90	90	90	90
Space group	*P*2_1_2_1_2_1_	*P*2_1_	*P*2_1_	*P*2_1_2_1_2_1_
Mosaicity (°)	0.316	0.180	0.306	0.120
Resolution range (Å)	48.63–2.34	47.70–2.74	45.88–2.80	62.53–3.25
Total no. of reflections	209,229	247,857	565,329	396,552
No. of unique reflections	29,308	36,012	35,284	26,861
Completeness (%)^*a*^	99.4 (96.7)	98.8 (93.2)	99.6 (96.1)	100.0 (100.0)
Redundancy	7.1 (7.1)	6.9 (6.7)	4.9 (4.4)	14.8 (15.3)
〈I/σ(I)〉	11.6 (3.3)	10.4 (1.6)	16.9 (1.1)	13.1 (1.0)
*R*_meas_	0.154 (0.624)	0.130 (0.910)	0.128 (1.542)	0.118 (2.774)
CC_1/2_ (%)	99.7 (50.6)	99.8 (87.4)	99.9 (65.3)	99.8 (60.9)
Solvent content (%)	52	57	56	68
Overall *B* factor from Wilson plot (Å^2^)	39	83	65	92
				
Refinement
Resolution range (Å)	48.63–2.34	47.70–2.74	38.38–2.80	62.52–3.25
No. of:
Protein atoms	3,900	8,063	8,213	6,144
Ligand atoms	124	124	124	97
Water molecules	72	62	60	
*R*	0.229	0.224	0.209	0.255
*R*_free_	0.261	0.269	0.254	0.318
RMSDs from ideal values (56)
Bond lengths (Å)	0.009	0.010	0.002	0.003
Bond angles (°)	1.27	1.18	0.53	0.69
Avg *B* factor (Å^2^)	40	74	100	153
				
MolProbity validation (50)
Clashscore	4.65	4.36	3.80	12.95
MolProbity score	1.96	2.01	1.49	2.22
Ramachandran plot
Favored (%)	96.1	97.0	96.4	87.9
Allowed (%)	3.9	3.0	3.6	10.5
Disallowed (%)	1.6
				
Protein Data Bank deposition
PDB code	6PH2	6PH3	6PPS	6PH4

a Values for the outer shell are given in parentheses: LOV-N13J21 C69S, 2.49 to 2.34 Å; LOV-PAS (dark), 2.90 to 2.74 Å; LOV-PAS (light), 2.85 to 2.80 Å; and LOV-PAS-HK (light), 3.47 to 3.25 Å.

10.1128/mBio.00264-21.1 FIG S1 Relevance of the regions flanking the LOV domain. (A) The crystal structure of the LOVN13J21 C69S construct is a parallel dimer. The N- and C-terminal flanking regions adopt an α-helical conformation (Nα and Jα helices, respectively). The Nα helix plays a central role in the dimer formation, interacting with the hydrophobic β-sheet. For comparison, the crystal structure of LOVJ5 (PDB code 3T50) is shown at the right, which forms an antiparallel dimer through interactions between the hydrophobic β-sheets. Darker tones indicate the regions involved in the dimer interface. (B) The N-terminal region of the LOV domain promotes the dimer formation. Size exclusion chromatography coupled to static light scattering (SEC-SLS) and dynamic light scattering (DLS) analysis of three LOV constructs containing only the LOV core (LOVJ5, residues 28 to 139), the LOV core plus the C-terminal flanking region (LOVJ21, residues 28 to 155) and the LOV core plus both the C- and the N-terminal flanking regions (LOVN13J20, residues 15 to 154) under dark conditions. The average and standard deviation values of the experimental molecular weights (MW) and hydrodynamic diameter (D_H_) are indicated, along with the expected MW and D_H_ of the monomers and dimers. The Nα but not the Jα helix is a key element for the parallel dimeric arrangement. (C) The presence of the N-terminal region of the LOV domain dramatically increases the half-life of the light state. Dark recovery rate followed by UV-Vis spectroscopy of the three LOV constructs mentioned above. The proteins were irradiated for 10 min with 10 μmol m^−2^ s^−1^ white light and then kept in the dark for 17 h, while the spectra were recorded. All experiments were performed at 25°C. Although full-length LOV-HK presents a stable light state (T. E. Swartz et al., Science 317:1090–1093, 2007, doi:10.1126/science.1144306), the isolated LOV core slowly decays to the dark state (in hours) (J. Rinaldi et al., J Mol Biol 420:112–127, 2012, doi:10.1016/j.jmb.2012.04.006). The presence of the Nα helix dramatically increases the half-life of the light state, while the Jα helix has no significant effect. Taken together, these results support the fact that the Nα helix plays an essential role in the formation of the parallel dimer, which presents an unusually slow dark recovery. Download FIG S1, PDF file, 2.8 MB. Copyright © 2021 Rinaldi et al. 2021 Rinaldi et al. https://creativecommons.org/licenses/by/4.0/ This content is distributed under the terms of the Creative Commons Attribution 4.0 International license.

Static light scattering experiments show that the LOV core constructs with and without the Jα helix (LOVJ20 and LOVJ5, respectively) present a monomer-dimer equilibrium. In contrast, the addition of the Nα helix (LOVN13J21 construct) strongly promotes the dimer formation (see Fig. S1B). In addition, the presence of the Nα helix dramatically increases the lifetime of the light state in LOVN13J21, with no detectable decay for at least 30 h after light excitation (see Fig. S1C and Fig. S2A), while the light states of both LOVJ5 and LOVJ20 slowly decay in hours (see Fig. S1C).

10.1128/mBio.00264-21.2 FIG S2 Comparison between LOV, LOV-PAS, and LOV-PAS-HK constructs. (A) Photoactivities of LOV (15 to 155), LOV-PAS (15 to 273), and LOV-PAS-HK (15 to 489) constructs. The absorption spectra were recorded immediately and 30 h after illumination. (B) Size exclusion chromatography coupled to static light scattering (SEC-SLS) of three constructs under dark and light conditions. The average and standard deviation values of the experimental MWs estimated by the relation of scattering/RI are indicated above each chromatogram, along with the expected MW of the monomer, below and after asterisks. For the LOV and LOV-PAS constructs a Superdex 75 column was used, while for the LOV-PAS-HK construct the gel filtration was performed in a Superdex 200 column. (C) Detail of the peaks shown in panel B. The solid, dashed, and dotted lines correspond to the scattering signal, the refractive index signal, and the calculated MW values, respectively. Taken together, the results indicate that the three constructs that contain the Nα helix present the same photochemical behavior and are dimeric. Download FIG S2, PDF file, 0.8 MB. Copyright © 2021 Rinaldi et al. 2021 Rinaldi et al. https://creativecommons.org/licenses/by/4.0/ This content is distributed under the terms of the Creative Commons Attribution 4.0 International license.

Taken together, these results (see Fig. S1 and Fig. S2) demonstrate that the Nα helix plays an essential role in forming a parallel dimer. Indeed, the inclusion of the Nα helix enabled us to crystallize and determine the crystal structures of this blue light photoreceptor in two multidomain constructs featuring different domain compositions (LOV-PAS and LOV-PAS-HK), including the full-length protein (35). In both constructs, the first 15 residues, which were predicted to be disordered, are excluded. More importantly, these constructs form photoactive and head-to-head, parallel dimers both in solution (see Fig. S2) and in their crystal lattices, as explained below.

### LOV-HK forms a parallel dimer structure via coiled-coil interactions.

We have determined the crystal structure of LOV-PAS (Fig. 2A; see also Fig. S3) in the dark-adapted state at 2.74 Å resolution (Table 1). The chromophore environment in the LOV-PAS structure is consistent with the dark structures of B. abortus LOV domains and other LOV proteins, where the FMN ligand is clearly separated from the protein moiety in the electron density map (see Fig. S4A, left). In the asymmetric unit, four LOV-PAS polypeptide chains form two elongated parallel dimers (see Fig. S3A). In each dimer, the LOV domains, the linker helices, and the PAS domains from the partner subunits are juxtaposed along the extensive dimer interface (Fig. 2A). The tandem LOV and PAS domains from the same subunit have no direct contact, and their dispositions are swapped relative to the helical spine resulting in a cross-shaped dimer. At the dimer interface, two long Jα helices (residues 135 to 171) consisting of five heptad repeats form an extensive coiled coil via mainly hydrophobic interactions.

10.1128/mBio.00264-21.3 FIG S3 Crystal structure, molecular packing and topology diagram of LOV-PAS. (A) Four LOV-PAS polypeptide chains form two nearly identical dimers related by translational noncrystallographic symmetry in the asymmetric unit (subunit A, yellow; subunit B, blue; subunit C, cyan; and subunit D, green). The AB and CD dimers are packed via an interface between subunits A and C. (B) Close-up view of the FMN chromophore in the LOV domain. (C) Topology diagram of LOV-PAS showing the Jα helix that connects the LOV and PAS domains. Download FIG S3, PDF file, 1.6 MB. Copyright © 2021 Rinaldi et al. 2021 Rinaldi et al. https://creativecommons.org/licenses/by/4.0/ This content is distributed under the terms of the Creative Commons Attribution 4.0 International license.

10.1128/mBio.00264-21.4 FIG S4 FMN binding site in the dark and light structures. (A) The FMN ligand and the conserved cysteine residue in the LOV domain (Cys69) of the dark LOV-PAS (left) and the light LOV-PAS-HK (right) structures and their electron density map contoured at the 1.0 σ level are shown. (B) Contacts of the FMN molecule and the residues Gln132, Asn101, and Asn111 in the dark LOV-PAS (left) and the illuminated LOV-PAS (right) structures. The conserved (black) and differential (red) distances are indicated. (C) Photoactivity in LOV-PAS crystals. A times series of difference (light-dark) absorption spectra obtained from a single-crystal of LOV-PAS during a 30-s period (from red to blue) under 450-nm light illumination showed the photobleaching of FMN, indicating the formation of the light state. Download FIG S4, PDF file, 2.1 MB. Copyright © 2021 Rinaldi et al. 2021 Rinaldi et al. https://creativecommons.org/licenses/by/4.0/ This content is distributed under the terms of the Creative Commons Attribution 4.0 International license.

We have also determined the crystal structure of LOV-PAS-HK in the light state at 3.25 Å resolution (Fig. 2B and Table 1). This full-length structure also shows a head-to-head, parallel scaffold from the N-terminal LOV domain to the C-terminal HK domain with a large surface area (10,240 Å^2^) buried at the dimer interface (Fig. 2B). Consistent with the light state of the LOV photoreceptors, the electron densities associated with the FMN chromophores are fused into the protein moiety in both LOV domains (see Fig. S4A, right). However, we were unable to discern the covalent linkage between the Cys69-Sγ and the FMN-C4a atoms likely due to the limited resolution. The nonhydrolyzable ATP analogue AMP-PCP (ACP) was added to the crystallization buffer to stabilize the HK domain. Only one of the two CA subdomains could be modeled due to the weak electron density. The calculated hydrodynamic diameter of the LOV-PAS-HK dimer is ∼110 Å, which agrees with the D_H_ value (121 ± 4 Å) determined from the solution scattering experiments (Table 2), taking into account that one CA subdomain is missing in the PDB coordinates. Compared to the largely straight LOV-PAS structure, the central helical spine in the full-length light structure consists of a series of helical bundles interrupted at different segments (Fig. 2B). While each modular domain dimerizes with its counterpart from the partner subunit, the LOV and PAS dimers are no longer coaxial, and their relative orientation dramatically differs from that in the LOV-PAS dark structure (Fig. 2A). As a result, the LOV-PAS-HK structure is a highly asymmetric dimer with an overall curvature of ∼150° pivoted around the PAS domain.

**TABLE 2:** Analysis of the intensity size distribution of LOV-PAS-HK samples

Sample	Mean ± SEM^*a*^				
	Z-avg (diameter, nm)	PdI	%Int_peak 1_	%Int_HMW_	D_H peak 1_ (nm)
LOV-PAS-HK light	26.17 ± 6.81	0.579 ± 0.090	58.3 ± 7.8	41.7 ± 10.5	12.96 ± 0.28
LOV-PAS-HK dark	13.93 ± 0.36	0.208 ± 0.035	94.2 ± 3.3	5.8 ± 4.5	13.28 ± 0.46
LOV-PAS-HK light + AMP-PCP	13.38 ± 1.07	0.227 ± 0.027	94.5 ± 7.8	5.5 ± 2.4	12.05 ± 0.39
LOV-PAS-HK dark + AMP-PCP	16.24 ± 7.55	0.221 ± 0.010	96.8 ± 1.9	3.2 ± 1.9	11.69 ± 0.19

a PdI, polydispersity index; % Int_peak_ _1_, % area of peak 1; %Int_HMW_, % area of high-molecular-weight (HMW) particles; D_H peak 1_, hydrodynamic diameter of the particles forming the peak.

**FIG 3:**
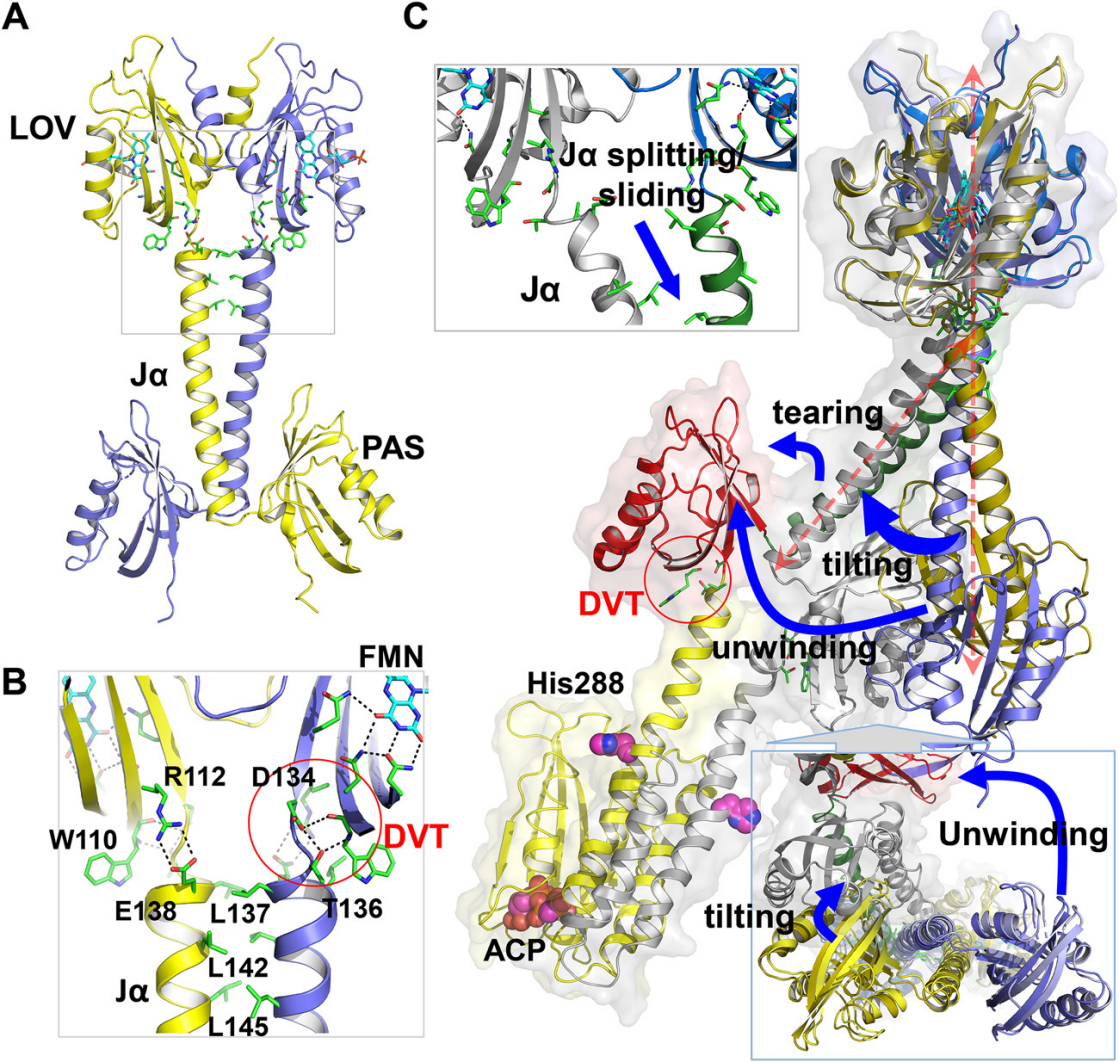
Structural comparison between LOV-PAS and LOV-PAS-HK. (A) Coupling between the LOV core and the helical spine in LOV-PAS. (B) A zoom-in view (from the gray box in panel A) highlights the conserved interactions from the FMN binding site to the coiled coil contacts between Jα helices. The FMN isoalloxazine ring is stabilized by a network of H-bonds at the chromophore site. The highly conserved Trp110 residue and the DVT sequence motif are located at the junction between the LOV core and the Jα helix. Two juxtaposed Jα helices are tethered via hydrophobic interactions mediated by Leu137, Leu142, and Leu145 at the dimer interface. (C) Alignment of the LOV-PAS dark structure (in blue/gold) and LOV-PAS-HK light structure according to the LOV dimer reveals a series of structural rearrangements. Blue arrows highlight the structural changes: Jα splitting, tilting of the helical spine, tearing of the PAS domain, and unwinding of the helical spine manifested in a 90° rotation of the PAS dimer. The magenta spheres represent the His288 phosphorylation site and the ATP-analogue molecule bound to the HK domain. The coupling between the PAS domain and the helical spine features a DVT/W motif similar to the LOV domain (red circle).

The LOV and PAS domains share nearly identical topology both in the LOV-PAS and full-length structures (see Fig. S3C). The LOV and PAS domains also share remarkable similarities in their modes of coupling to the central helical spine. In addition to the αβ core scaffold, both domains have an N-terminal helix packed against the β sheet in their corresponding partner subunits (see Fig. S3), while the C-terminal helices extending from the core domains form the coiled coil interactions at the dimer interface (Fig. 3A; see also Fig. S5C). At the junction between the core domain and the C-terminal helix, both the LOV and PAS domains feature a DVT sequence motif where the threonine residues (Thr136 in LOV and Thr270 in PAS) interact with the conserved Trp110 and Trp247 residues, respectively (Fig. 3B and C; see also Fig. S5B and G). Signal coupling between the core and the central helical spine is likely to be mediated by salt bridges such as Arg112-Glu138 and Lys103-Asp134 in the LOV domain and such as Arg192/Arg246-Asp268 and Glu249-Arg272 in the PAS domain (Fig. 3C; see also Fig. S5B and G).

10.1128/mBio.00264-21.5 FIG S5 Comparison of dark and light-adapted structures by region. (A) LOV domain; (B) LOV domain-Jα helix interface; (C) Jα helix coiled coil; (D) Jα helix-PAS domain interface; (E) relative orientation of the LOV and PAS domains; (F) PAS domain; (G) PAS domain-α1 helix of the HK domain interface; (H) HK domain. The monomers of the dark structure are colored in gray scale (lighter for chain A and darker for chain B), while the monomers of the light structure are colored in blue (chain A) and green (chain B). The red arrows indicate some of the light-gated perturbations that emerge from the comparison. The relative orientation between the LOV and PAS dimers in the dark and the light states is shown in a top view in panel E, in which the PAS domains are colored in red. In panel H, the “dark” HK domain corresponding to the dimer of the isolated HK crystal structure (PDB code 5EPV) is overlapped with the HK domain of the light structure. Download FIG S5, PDF file, 2.3 MB. Copyright © 2021 Rinaldi et al. 2021 Rinaldi et al. https://creativecommons.org/licenses/by/4.0/ This content is distributed under the terms of the Creative Commons Attribution 4.0 International license.

When the LOV-PAS and LOV-PAS-HK structures are aligned according to the LOV dimer framework (see Materials and Methods), a symmetric rotation of the LOV monomers within the dimer is observed (Fig. 3C; see also Fig. S5A). Also, the helical spine is significantly skewed in the Jα segment and tilted ∼60° from the straight spine of the LOV-PAS structure. Concomitantly, the PAS dimer in the full-length structure is rotated about 90° as if the helical spine were unwound (Fig. 3C; see also Fig. S5E), thereby placing the LOV and PAS domains from the same subunit on the same side of the dimer scaffold (Fig. 2A and B). Farther down into the HK domain, the helical spine evolves into a four-helix bundle via the DHp subdomain that brings the HK domains together (Fig. 2B). In contrast to the symmetry observed in the isolated B. abortus HK structure (PDB code 5EPV, Fig. 2C) (27), one of the linker helices in DHp displays a severe kink near residue Lys273, rendering a large bend in the helical spine of the LOV-PAS-HK structure (see Fig. S5H). In the full-length structure, the only CA subdomain that could be modeled adopts the same inactive conformation as in the isolated HK domain structure, with the ACP molecule bound to the active site located ∼30 Å away from the His288 phosphorylation site (see Fig. S5H) (27). The orientation of this CA subdomain and the DHp-CA interface are very similar in both structures (root mean square deviation [RMSD] = 1.29 Å for 174 aligned C^α^ atoms; residue range, 285 to 479). In the LOV-PAS-HK structure, the side chains of His288 in both chains are exposed to the solvent and in a similar location as in the crystal structure of the isolated HK domain in the inactive conformation. However, due to the low resolution of the full-length structure, the electron density corresponding to these side chains is very weak. The comparison of the DHp subdomains in both structures shows a slight rearrangement of the four-helix bundle (RMSD = 1.86 Å for 74 aligned C^α^ atoms; residue range, 285 to 334).

Taken together, both the dark LOV-PAS and the light LOV-PAS-HK structures adopt a parallel dimer scaffold with long linker helices tethered at the dimer interface. While the LOV-PAS dimer is largely straight and symmetric, the full-length protein adopts an elongated architecture with a tilted and crooked helical spine to which the juxtaposed globular domains (LOV, PAS, and CA) are attached.

### Dimer asymmetry is amplified from the N terminus to the C terminus in LOV-HK.

**FIG 4:**
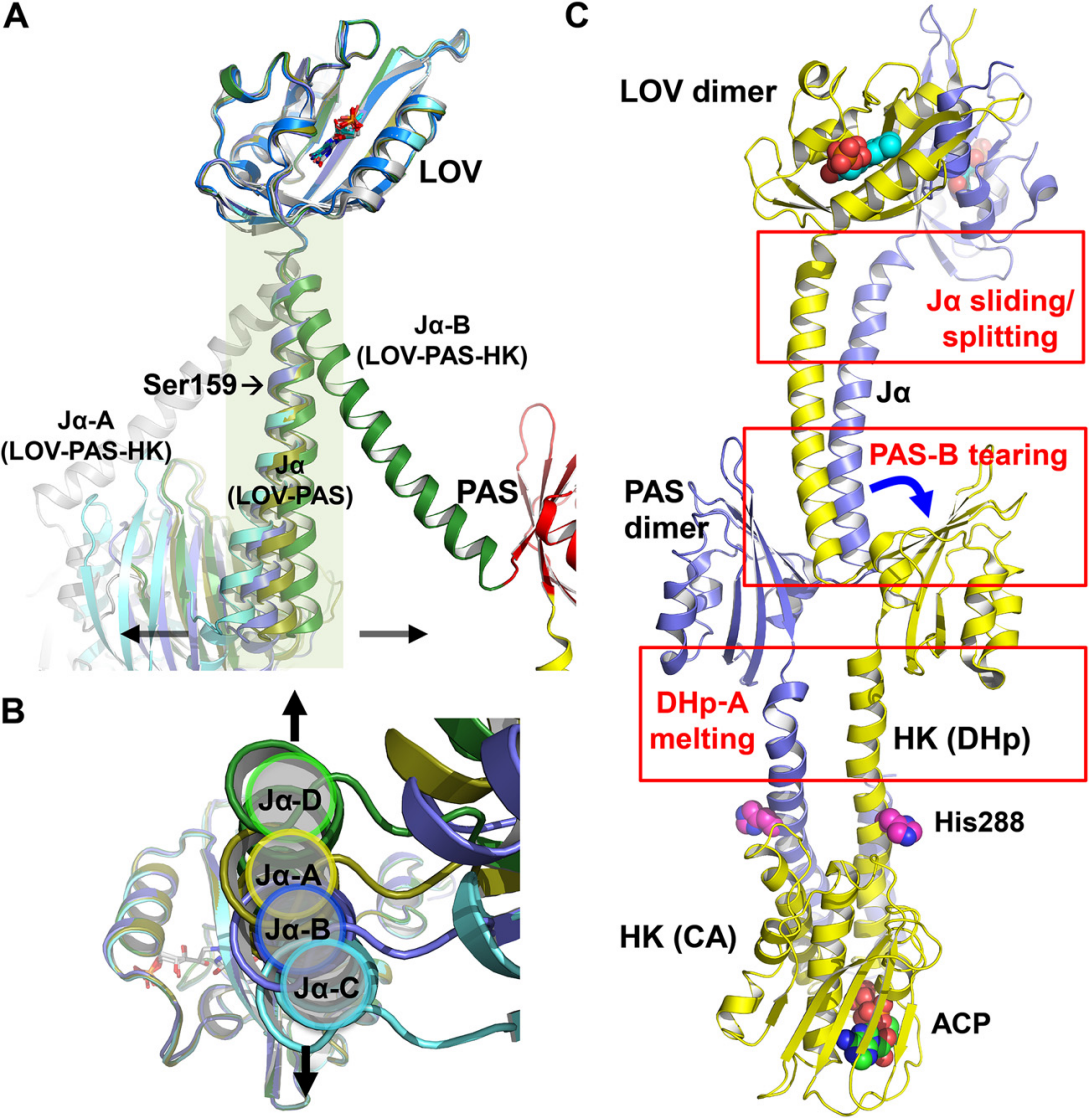
Dimer asymmetry in the LOV-PAS and LOV-PAS-HK structures. (A) The superposition of the monomer structures from LOV-PAS and LOV-PAS-HK according to the LOV core domain shows dimer asymmetry, which is much less marked in the LOV-PAS dimers than in the LOV-PAS-HK structure (dark green/gray). Subunits A, B, C, and D of LOV-PAS are colored yellow, blue, cyan, and green, respectively. (B) The bottom view of panel A shows small displacements of the Jα helices resulted from dimer asymmetry in LOV-PAS dimers, suggesting that the AB dimer is less asymmetric than the CD dimer. (C) Structural asymmetry in different segments of the LOV-PAS-HK dimer scaffold compared to the LOV-PAS structure.

Although the LOV-PAS structure is rather straight, modest dimer asymmetry is evident when the four monomers present in the asymmetric unit are aligned according to the LOV domain (Fig. 4A). The CD dimer exhibits slightly higher asymmetry than the AB dimer (Fig. 4B).

The full-length LOV-PAS-HK light structure, however, displays much more pronounced dimer asymmetry (Fig. 4A and C). When two subunits are aligned according to the LOV domains, the corresponding Jα helices diverge to completely different directions (Fig. 4A). In contrast to the dark LOV-PAS structure where the coiled coil interactions are mostly “in-register” or symmetric mediated by equivalent residues (Leu137A-Leu137B and Leu142A-Leu142B), the N-terminal half of the Jα helices engages new “off-register” or asymmetric interactions between residues shifted by one helical turn in the light LOV-PAS-HK structure (see Fig. S5C). These asymmetric interactions (Leu145A-Arg141B and Arg149A-Glu144B) suggest a relative sliding between the Jα helices, which concurs with the helical spine tilting relative to the LOV dimer (Fig. 3C and Fig. 4C). However, the C-terminal end of the Jα helices retains the “in-register” coiled coil contacts (Leu163A-Leu163B, Ile166A-Val167B, and Val167A-Ile166B). As a result of sliding, the Jα-A helix is kinked around residue Ser159 while the Jα-B helix remains largely straight (Fig. 4C), and the side chain of Arg158A loses its contacts with the H-I loop from PAS-B (see Fig. S5D). Such asymmetry is further amplified in the dimer scaffold as the protein chain moves toward the C-terminal end. First, the core β-sheet of PAS-B is torn away from Jα-A, possibly as a consequence of the loss of the contacts mentioned above, while PAS-A remains attached to the Jα-B (see Fig. S5D and F). Second, as the helical spine enters the HK domain, one of the parallel DHp helices (DHp-A) becomes melted or deformed, while DHp-B retains its α-helical conformation (Fig. 4C; see also Fig. S5H). Third, the CA subdomain of chain A is completely disordered, possibly resulting from enhanced conformational dynamics and/or flexibility of HK-CA in the activated state (see Fig. S5H).

### Light-induced structural changes originating in the LOV domain lead to dimer asymmetry.

Comparisons between the dark LOV-PAS and light LOV-PAS-HK structures suggest that the light activation of LOV-HK is accompanied by global concerted structural rearrangements as the protein transitions from a largely symmetric dimer to a highly asymmetric light state. To address whether these differences are indeed light-induced structural changes or whether they are simply due to different constructs crystallized under different conditions, we conducted dynamic crystallographic experiments to examine the light-induced structural changes in the photoactive LOV-PAS crystals. Our single-crystal spectroscopy experiments showed that the LOV-PAS crystals are indeed photoactive (see Fig. S4C). We collected 22 crystallographic data sets by subjecting the LOV-PAS crystals to various dark and light conditions at room temperature before freezing. Although the dark and light crystals belong to the same space group, they show small yet consistent differences in their cell parameters (see Fig. S6C). To detect the light-induced signals localized to the chromophore region, we applied singular value decomposition (SVD) to jointly analyze a collection of 88 (22 data sets × 4 subunits) simulated annealing omit maps (SAOMs) for which FMN and its adjacent conserved residues (131 to 134) were omitted. All SAOMs were aligned according to the rigid protein framework of the LOV domain based on the distance matrix analysis. The SVD analysis in real space is very effective for isolating subtle signals from those artifacts arising from nonisomorphism and/or model bias (36).

10.1128/mBio.00264-21.6 FIG S6 Light-induced global structural changes in LOV-PAS. (A) Light-induced structural changes in the LOV domain show partial domain separation at the junctions of the LOV cores and Jα helices. Red arrows represent the directions and amplitudes (×3) of the Cα displacements (if > 1Å) pointing from the dark to the light structure. (B) Light-induced structural changes in the Jα helical spine in the LOV-PAS dimer. The helical spine tilts in a direction perpendicular to the separation direction of the LOV domains. The block arrow marks the viewpoint for panel A. (C) Changes in unit cell parameters (normalized according to a) between the dark-adapted (black dots) and illuminated (blue dots) crystals suggest small light-induced expansion in the b and c axes. Download FIG S6, PDF file, 2.2 MB. Copyright © 2021 Rinaldi et al. 2021 Rinaldi et al. https://creativecommons.org/licenses/by/4.0/ This content is distributed under the terms of the Creative Commons Attribution 4.0 International license.

**FIG 5:**
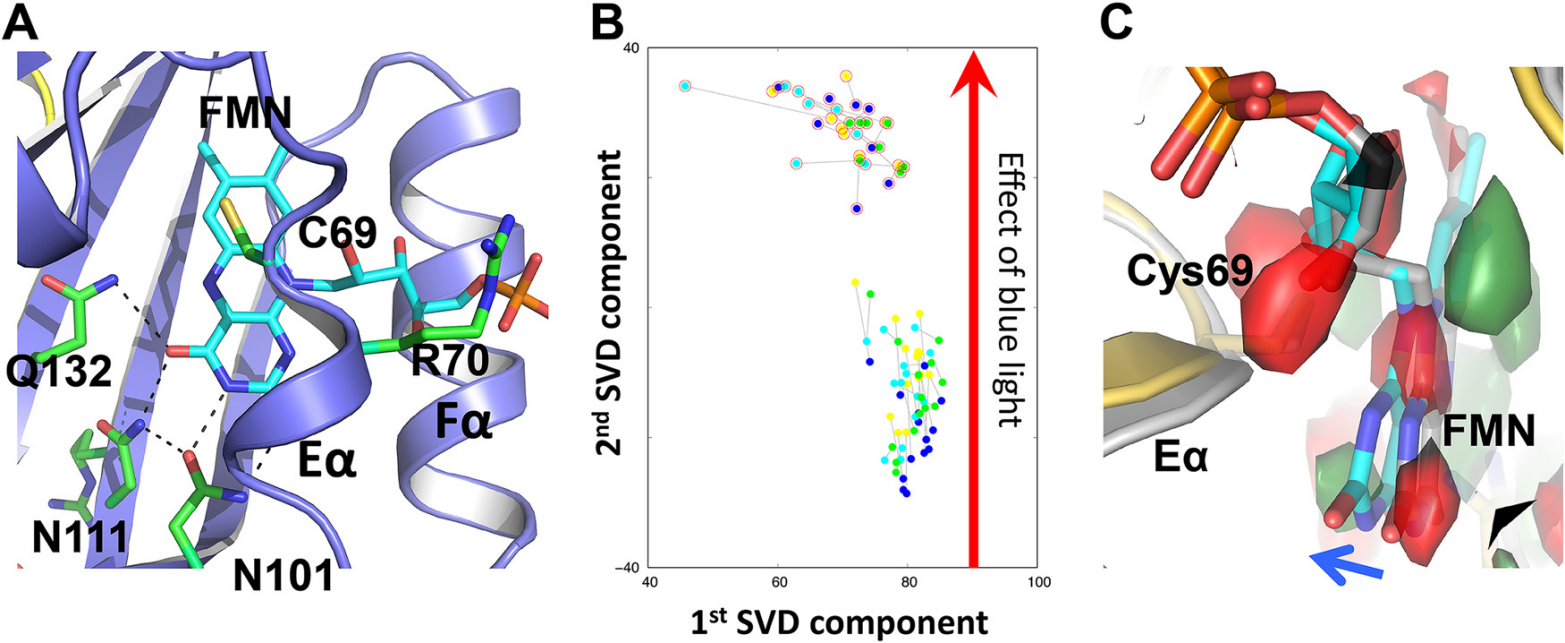
Light-induced tilting in FMN. (A) In the chromophore site, FMN is stabilized by the conserved residues Asn101, Asn111, and Gln132 with its phosphate group extending out between the Eα and Fα helices. (B) A scatterplot of the top two components from the SVD analysis of 88 simulated annealing omit maps of LOV-PAS near FMN reveals the light-induced signals between 14 dark data sets and 8 light data sets. Each dot corresponds to a map from subunit A (yellow), B (blue), C (cyan), and D (green). The light maps are highlighted with red circles. (C) The decomposed electron density map corresponding to the second component (green, positive density; red, negative density) clearly shows FMN tilting toward Eα upon blue light illumination. The two representative coordinates of dark-adapted (gray) and illuminated (colored) states shown were chosen from the SVD analysis.

In the SVD scatterplot corresponding to the top two components, the light and dark data sets are well separated along the second SVD dimension (Fig. 5A and B). The corresponding decomposed SVD map reveals the difference electron densities suggesting a consistent light-induced tilting of the FMN isoalloxazine ring (Fig. 5C). In the dark LOV structure, the isoalloxazine moiety of FMN forms extensive H-bonds with the conserved Asn101/Asn111 and Gln132 residues, while its phosphate group extends out between the Eα and Fα helices (see Fig. S4B). In the light LOV-PAS structure, all four subunits exhibit the tilting of FMN toward the Eα helix forming stronger H-bonds with Gln132 (see Fig. S4B). It is highly possible that the FMN tilting (see Fig. S7) results from the widely reported light-induced adduct formation between the FMN-C4a and Cys69-Sγ atoms, which is not fully resolved in our electron density maps.

10.1128/mBio.00264-21.7 FIG S7 Light-induced FMN tilting in the LOV-PAS structure. (A) Three regions in the FMN structure that were subjected to analysis. (B) The FMN binding site viewed from the tail region shows the tilting in the FMN ring plane upon light illumination. (C) “Light-minus-dark” difference distance matrix between the FMN chromophore and the main chain Cα atoms in the LOV domain. Difference distances highlighted in the green box show that the blue part of the FMN ring tilts away from the Eα helix, while the red part gets closer to the Eα helix. Download FIG S7, PDF file, 2.2 MB. Copyright © 2021 Rinaldi et al. 2021 Rinaldi et al. https://creativecommons.org/licenses/by/4.0/ This content is distributed under the terms of the Creative Commons Attribution 4.0 International license.

**FIG 6:**
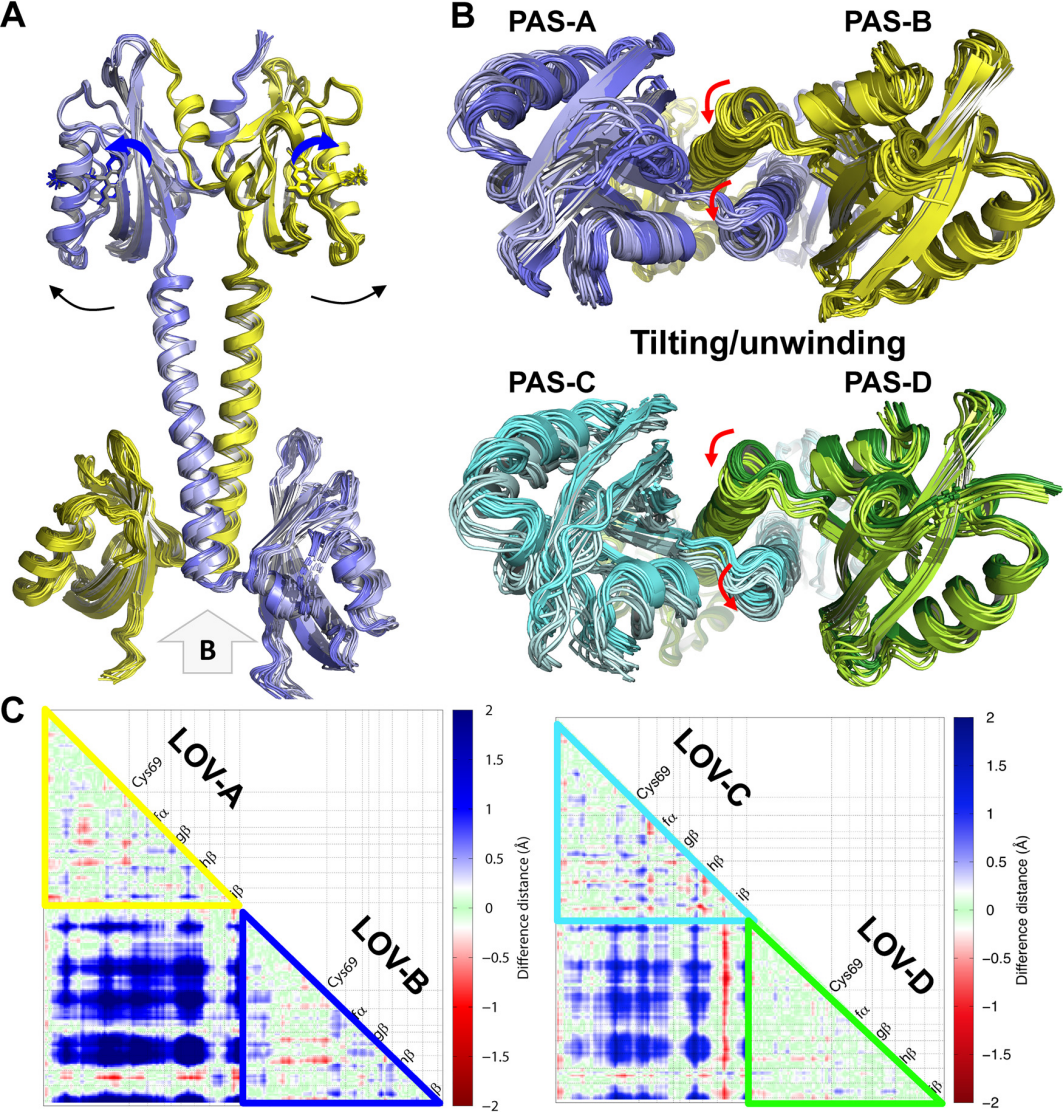
Light-induced protein structural changes in LOV-PAS. (A) Alignment of dark structures (in darker shade of yellow/blue) and light structures (in lighter shade of yellow/blue) of LOV-PAS. As FMN tilts outward (blue arrows), the C-terminal ends of the juxtaposed LOV domains partially separate (black arrows). (B) A bottom view of panel A shows that the tethered PAS dimer moves to the same direction (red arrows) in both AB (top) and CD (bottom) dimers. (C) The light-minus-dark difference distance matrix in the LOV domains of AB (left) and CD (right) dimers shows that the distances between the juxtaposed LOV domains increase by 0.5 to 2.0 Å (difference distances are color coded) upon blue light illumination. The LOV domain moves as a rigid body as the intradomain distances remain largely unchanged (small difference distance colored in green). The difference matrices are generated using utilities implemented in dynamiX (36). Both axes of the distance matrix plot represent the residue number of a corresponding LOV domain. In other words, the residue numbers in the *x* axis from left to right are exactly the same as those in the *y* axis from top to bottom. For clarity, the LOV domains are labeled along the diagonal of the distance matrix according to their secondary structures (as defined in Fig. S3C) *in lieu* of the residue numbers on both axes. Cys69 marks the position of the signature Cys residue in the FMN pocket of the LOV domain.

In addition to the FMN tilting, the LOV domain moves concertedly as a rigid body in response to light. Specifically, the LOV domains undergo partial separation across the dimer interface in both the AB and CD dimers (Fig. 6A; see also Fig. S6A), which is also evidenced by the increased intersubunit distances in the light-minus-dark difference distance matrices (Fig. 6C). We speculate that these light-induced protein structural changes are coupled to the FMN tilting via the conserved H-bonding interactions between FMN and Gln132 from the Iβ strand in the LOV core domain (Fig. 5A). Partial separation between the LOV domains results in a destabilized or altered dimer interface between the N-terminal parts of the Jα helices (Fig. 6A; see also Fig. S6B).

We also found that the light-induced structural responses in the helical spine are asymmetric, despite both LOV domains being torn apart in a symmetric manner (Fig. 6A). The helical spine remains intact by the extensive coiled-coil interactions at the dimer interface. Instead of moving with their respective LOV domains, the tethered Jα helices in both the AB and CD dimers move together sideways perpendicular to the helical spine, resulting in asymmetric responses between the partner subunits (Fig. 6B; see also Fig. S8A and B). Although these motions observed in the LOV-PAS crystals are small in amplitude due to lattice restraints, they are compatible with the Jα tilting captured by the light LOV-PAS-HK structure (Fig. 3C), where the helical tilting is coupled to unwinding of the helical spine, eventually leading to a large rotation of the PAS dimer relative to the LOV dimer (Fig. 3C). These dynamic crystallographic results strongly suggest that the major features captured by the light structure of LOV-PAS-HK are indeed induced by light, and they are allowed to fully develop in solution to adopt a highly asymmetric dimer conformation.

10.1128/mBio.00264-21.8 FIG S8 Flexibility and bending in Jα helices. (A) An RMSD distance matrix calculated from 36 LOV-PAS monomer structures shows that both LOV and PAS domains are relatively rigid with small RMSD values (<0.5 Å; marked in grey boxes). This rigid framework of the LOV domain is used as a reference for structural and/or map alignments. (B) Alignment of the monomer structures of LOV-PAS and LOV-PAS-HK shows displacement in the PAS domain due to differential bending of the Jα helices. The black arrows indicate the effect of Jα bending. (C) Interhelix angle between two segments of the same Jα helix calculated using helix parameterization. Jα-seg1, residues 150 to 159; Jα-seg2, residues 160 to 170. In the AB dimer, Jα-B is slightly more bent than Jα-A, while in the CD dimer, Jα-C is more bent than Jα-D. Download FIG S8, PDF file, 2.0 MB. Copyright © 2021 Rinaldi et al. 2021 Rinaldi et al. https://creativecommons.org/licenses/by/4.0/ This content is distributed under the terms of the Creative Commons Attribution 4.0 International license.

## DISCUSSION

### Allosteric activation mechanism of LOV-HK.

Our experimental data obtained by crystallographic and solution studies suggest that LOV-HK undergoes global structural changes in response to light. Dynamic and static light scattering experiments coupled to size exclusion chromatography (SEC) showed that light illumination does not alter the dimer stability of LOV-PAS-HK but leads to a more compact dimer evidenced by a moderate increase in the SEC elution volume in the light state (Table 2; see also Fig. S2B). This observation may also explain why we were able to crystallize LOV-PAS-HK in the light state but not in its dark state.

**FIG 7:**
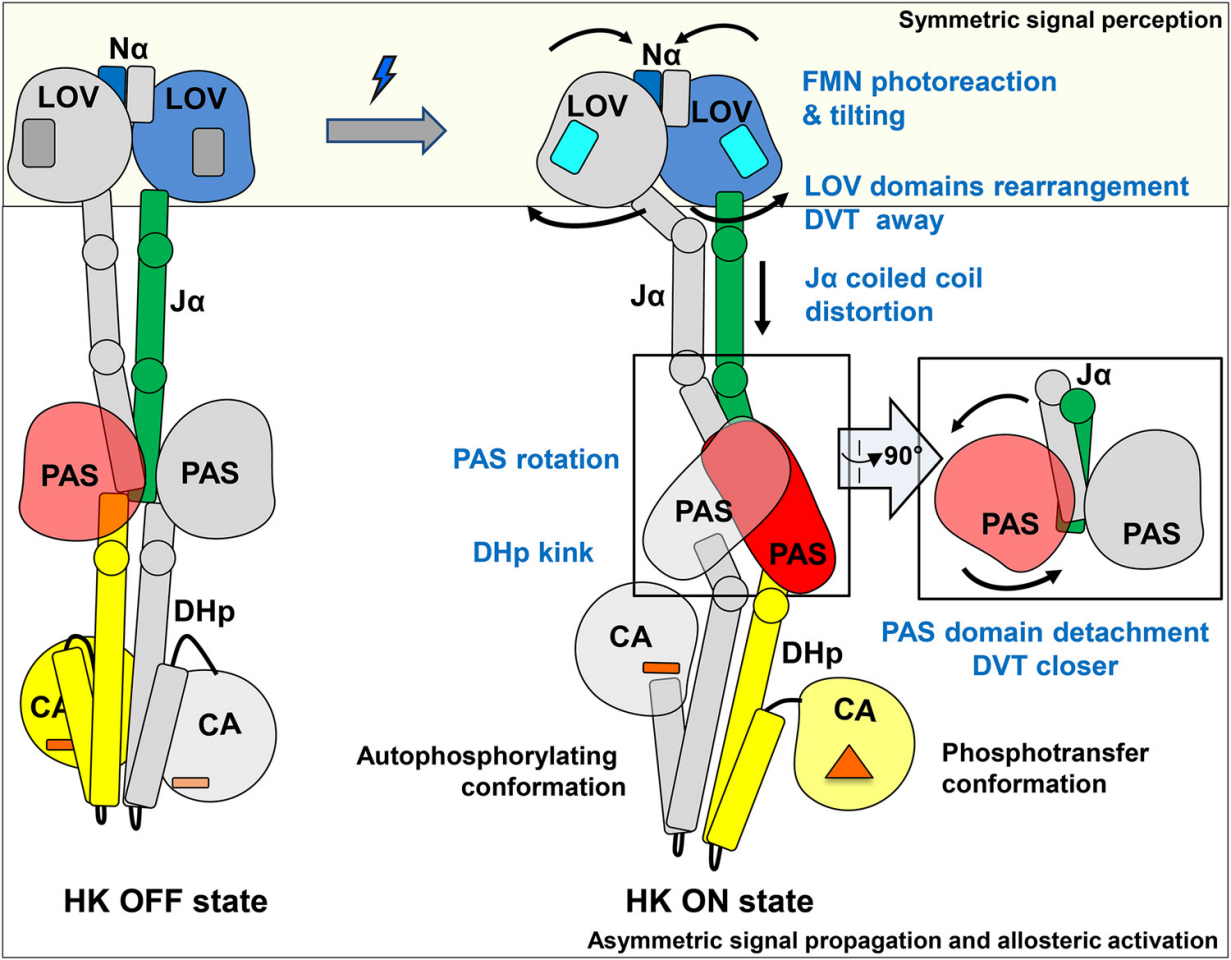
Allosteric activation mechanism proposed for SHK photoreceptors. The dark (left) and light (right) models are represented schematically and colored with the same color code used in Fig. 2. In the box on the right a side view of the PAS dimer in the light state is also shown. We propose a mechanism in which symmetric conformational changes within the LOV domains related to the light signal perception convert into asymmetric transitions related to signal propagation and allosteric activation. Squares, FMN; bars, ADP; triangle, ATP.

Based on comparisons between the dark and light structures, we postulate that light signaling in LOV-HK involves a series of structural events (Fig. 7), which starts with light-induced FMN photoreaction and tilting in the chromophore pocket. Concertedly, the distal end of the LOV domain undergoes a rotation relative to the central helices while the structural elements directly coupled to the Jα helices separate from one another. This is evidenced by the increased distance between the Cβ atoms in the two pivotal Val135 residues from 11 Å in the dark structure to 17 Å in the light LOV-PAS-HK structure and is consistent with the observations in other LOV proteins (20, 21, 33). This separation alters the coupling between the LOV core and the Jα helix. In LOV-PAS-HK, the coiled coil interactions mediated by Leu137 and Leu142 are torn apart, giving in to the “off-register” or asymmetric interactions in the N-terminal segment of the Jα helix, while the C-terminal portion remains unchanged.

These local changes in the LOV domain impose a conformational strain in the tethered helical spine. Relaxation of this strain drives further long-range asymmetric responses in the dimer scaffold of LOV-HK. First, the helical spine tilts sideway perpendicular to the separation direction of the LOV dimer. Second, the helical spine unwinds, leading to a nearly 90° rotation of the PAS dimer with respect to the LOV dimer. Third, one of the PAS domains is detached from the partially unwound helical spine, while the C-terminal ends of both PAS monomers move closer with a shorter distance between the Cβ atoms of Val269 in the DVT motif of 14 Å in the light state compared to 23 Å in the dark. Fourth, the strain caused by the PAS dimer rearrangement leads to a severe kink in one of the two DHp helices of the HK domain (Fig. 7), thereby activating the accompanied CA subdomain that is too dynamic to be observed. Such asymmetric bending has also been reported for other SHKs, including VicK (34), CpxA (29, 37), HK853 (38), KinB (39), and WalK (40).

We postulate that the helical tethering strategy manifested in LOV-HK is general for long-range actions or allosteric activation in signaling proteins, including SHKs of similar modular architecture. Similar to a “spring-loaded trigger” mechanism, the energy that drives long-range structural changes is inherent to the protein quaternary structure held together by the coiled-coil interactions between long linker helices, whereas light-induced FMN tilting just serves as a trigger. Key to this signaling strategy is the plasticity of long helices that can bend, slide, tilt, unwind, stretch, or compress, allowing the juxtaposed helices from different subunits to adopt different, yet complementary conformations or curvatures and the DVT/W motifs at the N-terminal ends of these α helical elements. For example, in the light LOV-PAS-HK structure, Jα-B is clearly straighter than Jα-A, and the same is observed for the linker helices to the HK domain (Fig. 2B and Fig. 7). This dimer asymmetry is also evident in structural responses of modular domains coupled to the central helices. Although the LOV and PAS dimers share the same helical spine, the PAS dimer rotates in contrast to separation between the LOV monomers. Our helix parameterization shows that upon light illumination, the Jα helices move concertedly by complementing each other in helix curvature (see Fig. S8C). Specifically, although the bent helix is straightened, its counterpart becomes more bent (Fig. 7).

### Dimer asymmetry.

Modular signaling proteins employ various molecular strategies to alter dimer asymmetry, where the nature and amplitude of helical motions differ from system to system. While B. abortus LOV-HK undergoes an “off-register” rearrangement similar to those proposed for DesK (a membrane-bound HK from the HisKA3 family) (41) and other photoreceptors (42, 43), light activation of YF1 is facilitated by supercoiling of the tethered helices with a severely bent helical spine (20). In the phosphorylation-responsive photosensitive histidine kinase (PPHK), dimer asymmetry arises from differential coupling between the sensor domains to the helical spine (44).

It has been proposed that in the inactive or OFF state, SHKs are symmetric dimers and the α helices in the DHp subdomain are more or less straight (2). Upon activation to the ON state, the dimeric structure becomes more asymmetric with one subunit in the autophosphorylating conformation (or Michaelis complex) and the partner subunit in the phosphotransferase state in complex with the RR. In the ON state, the signaling helix in the DHp subdomain bends to allow the dimer asymmetry (2). In accordance, the light structure of LOV-HK in the active state is highly asymmetric, where the ordered CA subdomain adopts an inactive form, while the disordered CA subdomain enables *cis*-autophosphorylation. With such dimer asymmetry, a single phosphorylation event is expected to occur in each homodimer, as proposed for other HKs (29, 37, 45–47). As the autophosphorylation-capable monomer gets close to the PAS domain, the binding of the RR may not be allowed (Fig. 7; see also Fig. S5H in the supplemental material). The current hypothesis implies that the other monomer adopts the phosphotransferase conformation. In the LOV-PAS-HK crystal structure, the position of the PAS domain (chain B) is farther away from the ordered CA subdomain in the same chain, likely representing the phosphotransferase conformation (Fig. 7; see also Fig. S5H).

Some deviations from the “ON/asymmetric – OFF/symmetric” hypothesis are observed. For instance, a very recent study on CpxA (a bacterial SHK from the HisKA family) has shown that CpxA is diphosphorylated at both histidine sites of the homodimer, indicating that structural asymmetry observed in this protein may not be strictly related to the “half-site” activity in HisKA (48). Another example of divergence is VicK, where the helical bending of its DHp subdomains is required for its phosphatase activity (34). The latter observation contrasts with most of the SHKs, where the bending of the DHp subdomain is often associated with the kinase ON state (2).

Although the DHp bending is proposed as a general feature in the activation mechanism, members of the HisKA (HK853, CpxA, and EnvZ) and HisKA3 (DesK) families differ in the transition mechanism between the kinase and phosphatase states. In HisKA3, the transition between states seems to involve the rotation of the α1 helix, which positions the histidine residue for phosphorylation in the kinase state and alternatively occluding it in the phosphatase state. The histidine residue in the HisKA3 family does not participate in the phosphatase reaction (45, 47). In contrast, in HisKA the rotameric state of the phosphorylatable histidine residue is critical for the phosphatase activity, for which the histidine residue is essential (49–51). In B. abortus LOV-HK, the phosphorylatable histidine is solvent accessible. Structural comparisons between full-length LOV-PAS-HK and the truncated HK domain show no helical rotation or rotameric conformational changes in this residue. However, more structural information is needed in order to hypothesize on the transition between the kinase and phosphatase states in B. abortus LOV-HK.

In summary, we captured concerted light-induced structural changes from a largely straight dimeric structure in an inactive LOV-HK sensor in the dark state to a highly asymmetric bent structure in its activated state. We also identified the light-induced motions that give rise to this transition. Although light-induced responses in the light-sensing LOV domains are symmetric around the FMN chromophore, the slight asymmetry between the juxtaposed Jα helices observed in the dark state is significantly amplified via the modular architecture upon light activation, thereby altering the HK function. Using constructs featuring various domain combinations, we present here a detailed dissection of the signaling mechanism by which blue light perception in the LOV domain triggers local structural changes, which then propagate through the PAS domain and reach the output HK domain. These findings provide structural insights into the allosteric modulation of the signaling transduction in bacterial TCSs.

## MATERIALS AND METHODS

### Gene cloning and measurement of the intracellular level of phosphorylated PhyR.

B. abortus 2308 wild type (wt), the *lovhk* mutant (*lovhk*::*km*) (17), the *lovhk* mutant complemented with the pKS-*lovhk* plasmid (*lovhk*::*km* pKS-*lovhk*), and the *lovhk* mutant complemented with the pKS-*lovhk* C69S plasmid (*lovhk*::*km* pKS-*lovhk* C69S) strains were used. The recombinant pKS-*lovhk* plasmid was constructed by the restriction free cloning method (52). Briefly, the *lovhk* gene was amplified by PCR, including a fragment of ∼500 bp upstream of the start codon in order to include its native promoter, and the resulting fragment was used as a megaprimer for a PCR using the pKS vector as the template, obtaining the pKS-*lovhk* vector. The pKS-*lovhk* C69S plasmid was generated using the recombinant plasmid pKS-*lovhk* as the template, through a Gibson assembly approach (53). Briefly, two independent amplicons were generated from the template using specific primers (one set of primers bearing the specific mutation, while the other corresponds to the vector backbone). The two constructs were verified by DNA sequencing and introduced into the B. abortus 2308 *lovhk* mutant by conjugation with the Escherichia coli S17-1 strain. Bacteria bearing the pKS-*lovhk* plasmid were selected by resistance to nalidixic acid (B. abortus natural resistance), kanamycin, and chloramphenicol in tryptic soy agar plates.

The four strains were grown in tryptic soy broth rich medium under dark conditions until logarithmic phase (optical density at 600 nm [OD_600_] = 1.0). Under these favorable conditions, low initial levels of PhyR∼P are expected. This is especially important since PhyR could be phosphorylated by more than one HK sensor in response to different stress stimuli (13). Each strain was initially illuminated with a 1-s pulse of white light at 2,000 μmol m^−2^ s^−1^ or kept in the dark and then incubated for 10 min at 37°C under 42 μmol m^−2^ s^−1^ white light illumination or under dark conditions. After the illumination period, the bacteria were lysed concentrating the samples to one-tenth of the volume, and the amount of phosphorylated and unphosphorylated PhyR was quantified by Phos-tag gel electrophoresis and Western blotting with an anti-PhyR antibody (17). The volumes of the extracts were adjusted in order to load the same amount of cells, which were estimated by their OD_600_ values and checked by SDS-PAGE and Coomassie brilliant blue staining. The manipulation of the dark samples was performed under dim red light. All B. abortus strains used in this study were manipulated in a biosafety level 3 laboratory available at the Leloir Institute according to national regulations.

### Gene cloning, protein expression, and purification.

The gene cloning for all constructs was done applying a restriction-free strategy. Briefly, a first PCR was run using suitable primers to amplify the DNA region corresponding to the LOV-HK fragment, and the obtained fragment served as megaprimer in a second PCR with the pET-24a cloning vector as the template. DpnI was used to degrade the template DNA. The quality of the obtained constructs was assessed by DNA sequencing. The constructs bear a 6×His tag at their C termini. Escherichia coli BL21(DE3)/pLysS cells were transformed with the recombinant plasmids and grown overnight in Luria-Bertani (LB) medium added with 35 μg ml^−1^ kanamycin at 37°C with agitation (250 rpm). It is important to note that all of the following steps of protein production and purification were performed in the dark (dim red light), either in special adapted rooms or using laboratory glass material and other equipment covered with aluminum foil. Precultures were diluted in LB media or ZYM-5052 auto-inducing medium (54) and grown initially for 3 h at 37°C and then overnight at 18 or 28°C with agitation (200 rpm). Bacteria were centrifuged at 10,000 × *g* for 8 min at 4°C. The pellets were resuspended and sonicated in a solution consisting of 50 mM Tris, 0.5 M sodium chloride, 20 mM imidazole, 1 mM phenylmethylsulfonyl fluoride (PMSF), and 1 mM dithiothreitol (DTT; pH 7.4 to 8.2) (buffer A) and then centrifuged at 160,000 × *g* in a Beckman Coulter L7-65 ultracentrifuge (Brea, CA) for 1 h at 4°C. The supernatants were filtered through a 0.45-μm-pore-size membrane and loaded onto a HisTrap HP column (all columns were from GE Healthcare, Little Chalfont, England) connected to a Gilson FPLC apparatus (Luton, England). Elution was performed with a linear gradient of buffer B consisting of 50 mM Tris, 0.5 M sodium chloride, 0.5 M imidazole, 1 mM PMSF, and 1 mM DTT (pH 7.4 to 8.2). The appropriate protein fractions corresponding to the major peak were pooled and dialyzed overnight at 4°C against buffer C (50 mM Tris, 0.25 M sodium chloride, 1 mM PMSF, and 0.5 mM DTT [pH 7.4 to 8.2]) and further purified by gel filtration chromatography on Superdex 200 or Superdex 75 16/60 columns with isocratic elution in buffer C. A single peak was observed for all constructs. The selected protein fractions were then concentrated by centrifugation in Amicon Ultra-4 devices (Millipore, Billerica, MA). For UV-Vis spectroscopy and light scattering measurements, the samples were concentrated to approximately 1 to 3 mg ml^−1^. For the crystallographic studies, the samples were concentrated to 5 to 15 mg ml^−1^ and simultaneously exchanged into lower-ionic-strength crystallization buffer (10 mM Tris, 100 mM sodium chloride [pH 7.4 to 8.2]). The concentration of the samples was estimated by using the calculated molar extinction coefficient at λ = 280 nm provided by the ExPASy ProtParam tool based on the polypeptide sequence, subtracting approximately 25% of the total absorbance coming from the contribution of the FMN cofactor in the dark. For this purpose, an absorbance standard calibration curve of this ligand was used. The proteins were aliquoted, flash frozen in liquid nitrogen and stored at −70°C. The quality of the final preparations was assessed by SDS-PAGE, UV-Vis spectrophotometry, and static light scattering.

### Dark state recovery measurements in solution.

UV-Vis absorption spectra were collected every 30 min for 17 h at 20.0 ± 0.2°C on an Agilent Cary60 UV-Vis spectrometer (Santa Clara, CA) as described previously (4). The optical path length was 1 cm. The samples were illuminated with white light of 10 μmol m^−2^ s^−1^ fluence for 10 min prior to the measurements. The protein concentration was 70 μM. The buffer contained 20 mM Tris and 0.1 M sodium chloride (pH 7.0).

### Photobleaching measurements on single crystals.

Time series of difference absorption spectra on single LOV-PAS crystals were recorded during a 30-s period using a microspectrophotometer under a 450-nm pump light. This microspectrophotometer is equipped with an optical lens system with ×100 magnification coupled to a high-sensitivity spectrometer (QEPro; Ocean Optics, Largo, FL), which enables accurate measurements from a sample (solution or single crystal) with an optical surface as small as 25 μm.

### Size exclusion chromatography and static light scattering measurements.

The average molecular weight (MW) of LOV-HK in solution was determined on a Precision Detector PD2010 90° light scattering instrument (Bellingham, MA) tandemly connected to a high-performance liquid chromatography and an LKB 2142 differential refractometer. The columns used were Superdex 75 and 200 GL 10/300. Then, 250 μl of each purified protein at 20 μM was injected into the column, and chromatographic runs were performed with buffer containing 50 mM Tris-HCl and 0.25 M sodium chloride (pH 8.2) under isocratic conditions at a flow rate of 0.4 ml min^−1^ at 20°C. The MW of each sample was calculated by relating its 90° scattering and refractive index (RI) signals. Data were analyzed with the Discovery32 software supplied by Precision Detectors. The averages and standard deviations correspond to the central 10% of the peak.

### Dynamic light scattering measurements.

Size distribution and hydrodynamic diameter (D_H_) measurements were performed at 25°C with a Zetasizer Nano-S apparatus (Malvern Instruments, Malvern, United Kingdom) using a low-volume quartz cuvette. Protein samples were diluted to 1 to 2 mg ml^−1^ in 10 mM Tris-HCl and 100 mM sodium chloride (pH 8.2). For the particular case of LOV-PAS-HK, a 10-min incubation in white light (10 μmol m^−2^ s^−1^) or darkness was performed, and the presence or absence of AMP-PCP (ACP) and MgCl_2_ (both 3 mM) was also tested. For each sample, 10 runs of 10 s were performed. The size distribution by intensity and hydrodynamic diameters were calculated using the multiple narrow distribution analysis model of the DTS v.7.11 software (Malvern Instruments).

### Protein crystallization.

Initial crystallization conditions were screened at room temperature in 96-well sitting-drop vapor diffusion Greiner 609120 plates (Monroe, NC) using a Honeybee963 robot (Digilab, Marlborough, MA) and crystallization kits from Jena Bioscience (Jena, Germany) and Hampton Research (Aliso Viejo, CA). The following protein concentrations were used: 8.0 mg ml^−1^ (LOV-N13J21 C69S), 15.0 mg ml^−1^ (LOV-PAS), and 5.3 mg ml^−1^ (LOV-PAS-HK). For the latter protein, a complete description of the sample preparation, manipulation, and data collection has been presented elsewhere (35). Crystallization conditions were optimized using the following solutions: 15% (wt/vol) PEG 3350, 0.1 M sodium citrate (pH 5.2; N-LOV-C69S), 14% (wt/vol) PEG 4000, 0.2 M lithium sulfate, and 0.1 M Tris-HCl (pH 7.5; LOV-PAS). The LOV-PAS crystals were grown in the dark and manipulated under dim red light. All crystal samples were cryo-protected in their respective mother liquors added with PEG 400 or 2-methyl-2,4-pentanediol (MPD) and then flash cooled in liquid nitrogen using Hampton Research loops.

### Data collection, structure determination, and refinement.

X-ray diffraction data collection was performed at Synchrotron SOLEIL (France), as detailed in Table 1. All structures were solved by the molecular replacement method using the coordinates of the LOV-core domain in the dark (PDB code 3T50 [25]) and the isolated HK domain (PDB code 5EPV [26, 27]) when necessary. LOV-N13J21 C69S was the first structure solved, and then it was used as a template to solve LOV-PAS in the dark. The resolution of the light LOV-PAS-HK structure required a more complex protocol that has been already published (35). Manual building was performed in all cases with COOT (55), whereas refinement was done with Phenix.refine (56). The final models were validated with MolProbity (57) and deposited in the Protein Data Bank, as indicated in Table 1.

### Crystallization and structure determination for dynamic crystallography.

The dark-adapted LOV-PAS crystals were grown in the dark under crystallization conditions (14% [wt/vol] PEG 4000, 0.2 M lithium sulfate, and 0.1 M Tris-HCl [pH 7.5]) in a 1:1 ratio of protein (14 mg ml^−1^) and reservoir solution using the hanging-drop vapor diffusion method. Crystals of the typical size 80 × 80 × 150 μm^3^ appeared as a cluster in 3 to 5 days. Single crystals were harvested under red safety light for storage in liquid nitrogen before data collection.

The blue light illuminated LOV-PAS crystals were obtained by preilluminating the native crystals for 15 to 20 min by filtered blue light (450 nm) at room temperature to initiate the photoreaction. Then, X-ray diffraction data sets were collected at the Life Science Consortium Access Team Sector 21 of the Advanced Photon Source, Argonne National Laboratory. All diffraction images were indexed, integrated, and scaled using HKL2000 (58). The deposited crystal structure of light LOV-PAS (Table 1) was determined in the *P*2_1_ space group by the molecular replacement method using the previously determined LOV-PAS structure (PDB code 6PH3) and refined with Phenix.refine (56).

### Analysis of the coordinates and maps.

COOT (55), Bendix in VMD (59), and PyMOL (60) were used for analysis and illustration. The buried area of LOV-PAS-HK was calculated by the PISA server (61). The hydrodynamic diameter estimation from the LOV-PAS-HK coordinates was performed with the HullRad server (62). The joint analysis of the dynamic crystallography data sets was carried out using the published method (dynamiX) (36). For this analysis, we used a LOV dimer framework comprising the following residues belonging to the core of the LOV domains (C^α^ atoms): 22 to 41, 44 to 60, 63 to 71, 94 to 102, 113 to 119, and 126 to 130.

### Data availability.

The X-ray crystallographic coordinates and structure factor amplitudes reported in this work have been deposited at the Protein Data Bank under the following codes: LOV-N13J21 C69S, 6PH2; LOV-PAS (dark), 6PH3; LOV-PAS (light), 6PPS; and LOV-PAS-HK (light), 6PH4.
