# Supplementary material for: Dimer Asymmetry and Light Activation Mechanism in Brucella Blue-Light Sensor Histidine Kinase
Source: mBio. 2021 Apr 20;12(2):e00264-21. doi: 10.1128/mBio.00264-21 (PMC8092228; doi:10.1128/mBio.00264-21)

A

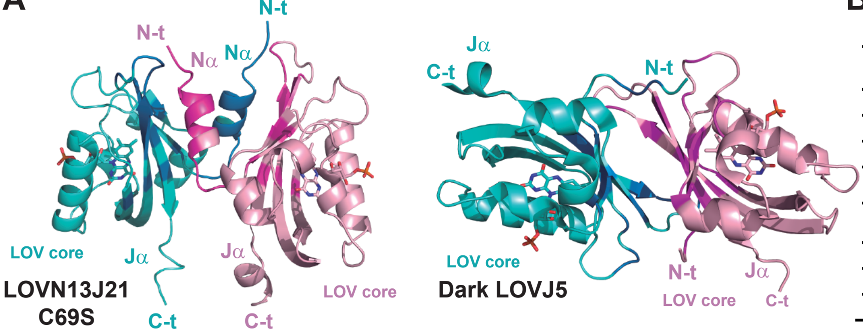

B

Oligomeric state of LOV-containing constructs

Constructs	Monomer MW (kDa)	Dimer MW (kDa)	SLS MW (kDa)
LOVJ5 (28-139)	14.4	28.8	21 ± 2
LOVJ20 (28-154)	15.4	30.8	20 ± 1
LOVN13J21 C69S (15-155)	16.7	33.4	34 ± 2

Protein concentration: 0.06 mM; Volume injected: 500 μl

Constructs	Monomer D_H (nm)	Dimer D_H (nm)	DLS D_H (nm)
LOVJ5 (28-139)	3.88	4.74	3.49 ± 0.24
LOVJ20 (28-154)	3.94	4.82	4.12 ± 0.30
LOVN13J21 C69S (15-155)	4.05	4.95	5.35 ± 0.39

Protein concentration: 0.06 mM

C

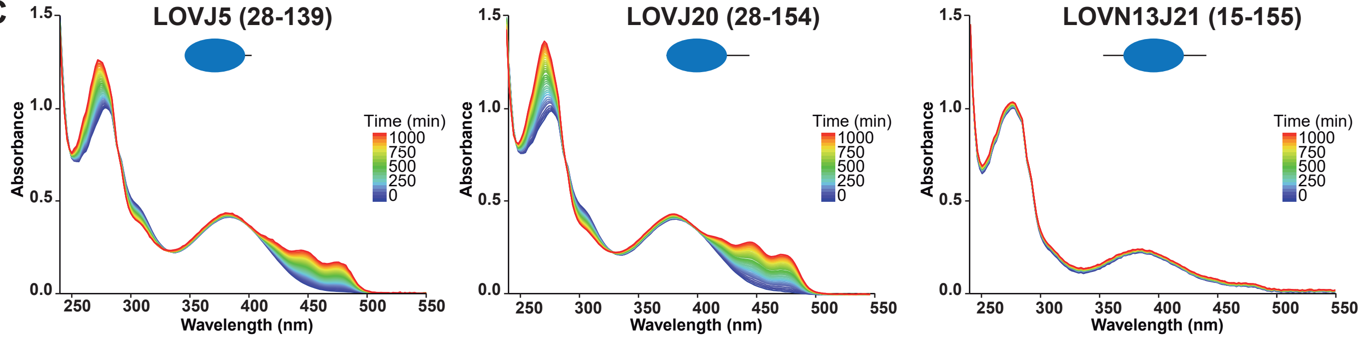

Supplement: FIG S1 [file mBio.00264-21-sf001.pdf]
