# Supplementary figures and images for: Dimer Asymmetry and Light Activation Mechanism in Brucella Blue-Light Sensor Histidine Kinase
Source: mBio. 2021 Apr 20;12(2):e00264-21. doi: 10.1128/mBio.00264-21 (PMC8092228; doi:10.1128/mBio.00264-21)

**A****LOVN13J21 (15-155)**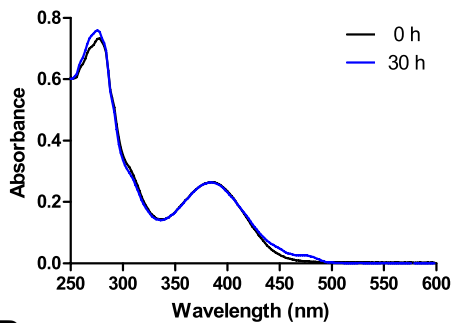**LOV-PAS (15-273)**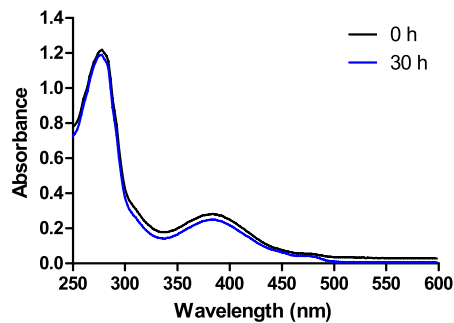**LOV-PAS-HK (15-489)**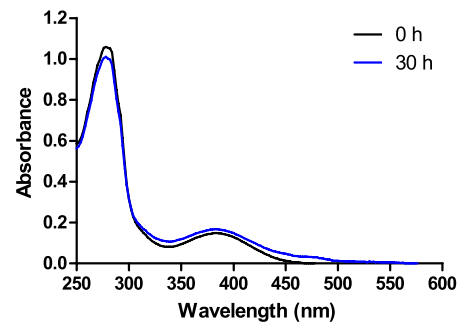**B**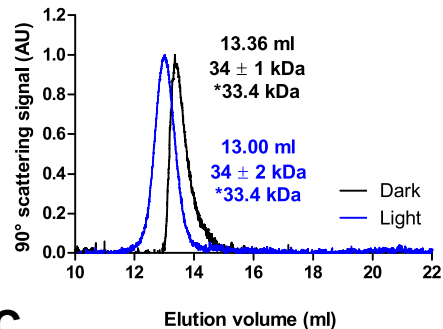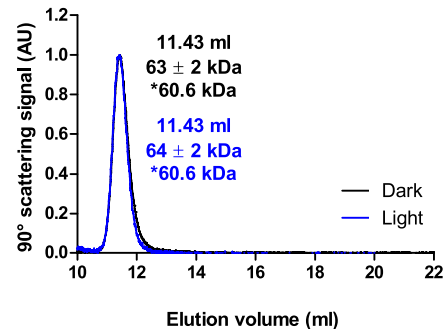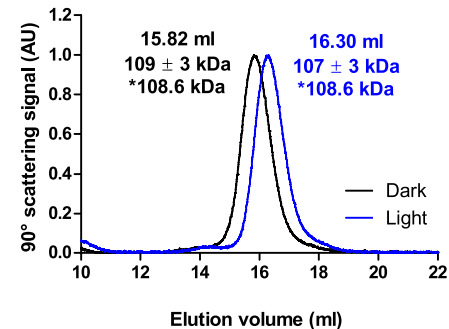**C**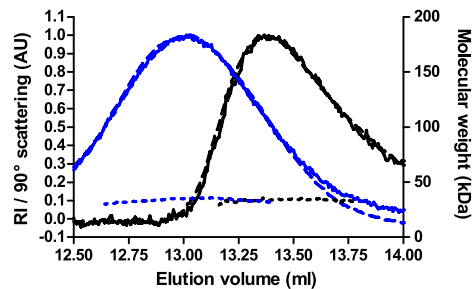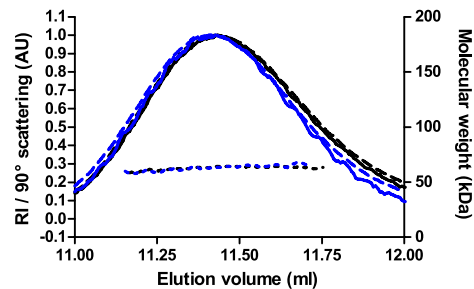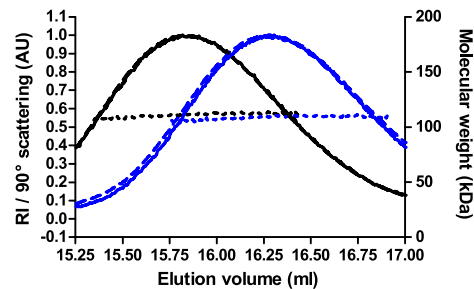

Supplement: FIG S2 [file mBio.00264-21-sf002.pdf]

**A**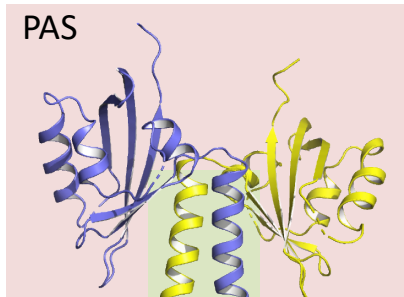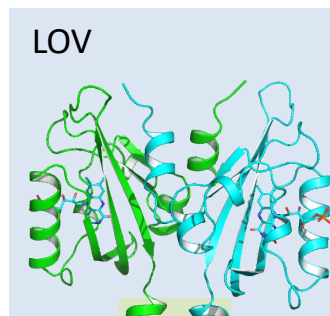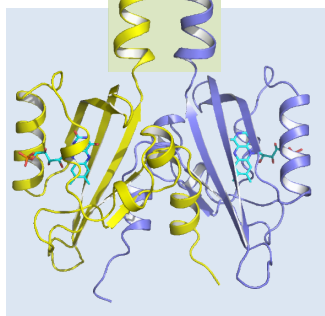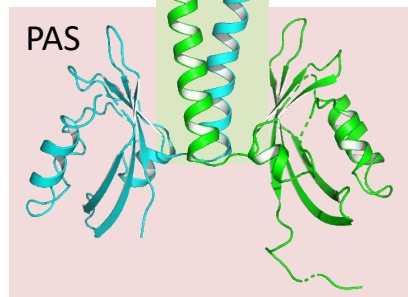**B**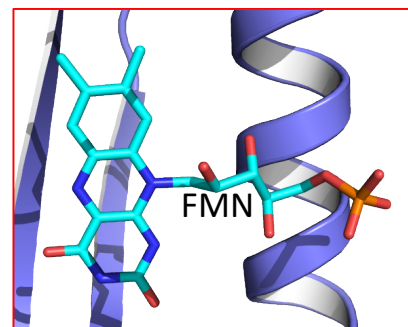**C**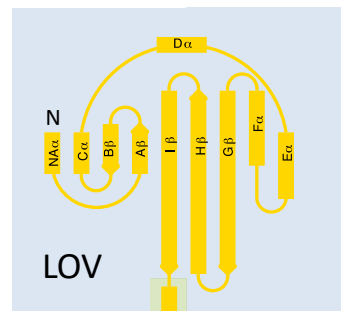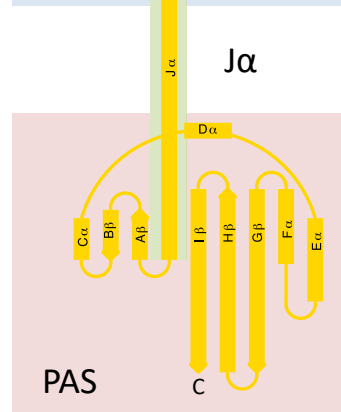

Supplement: FIG S3 [file mBio.00264-21-sf003.pdf]

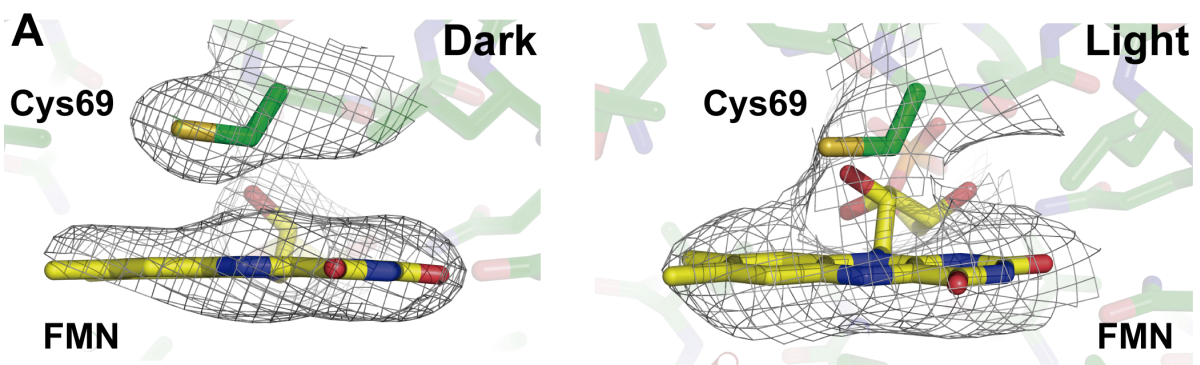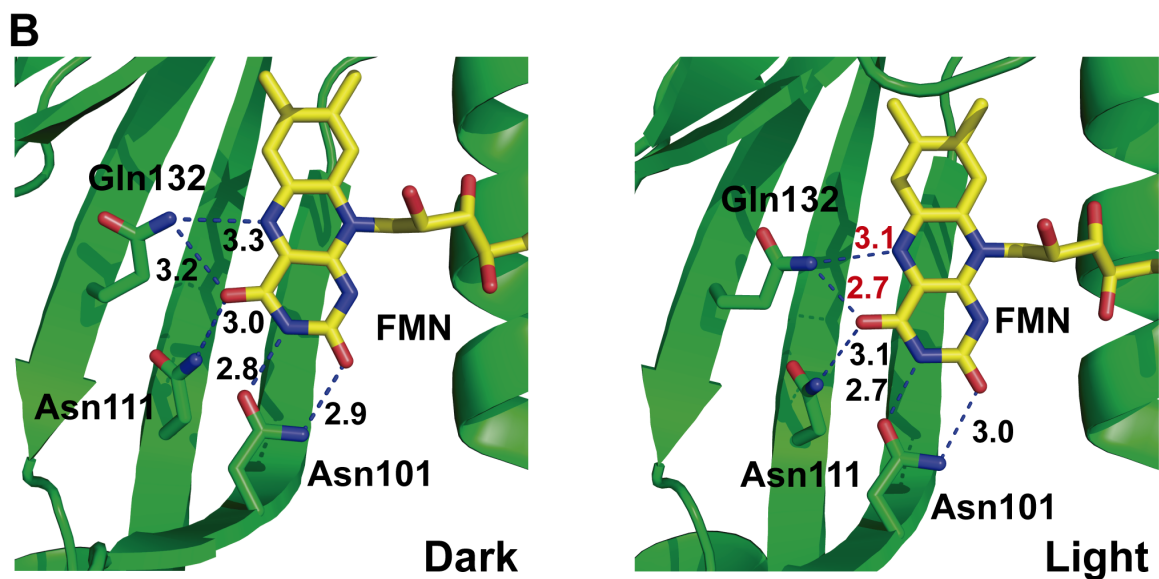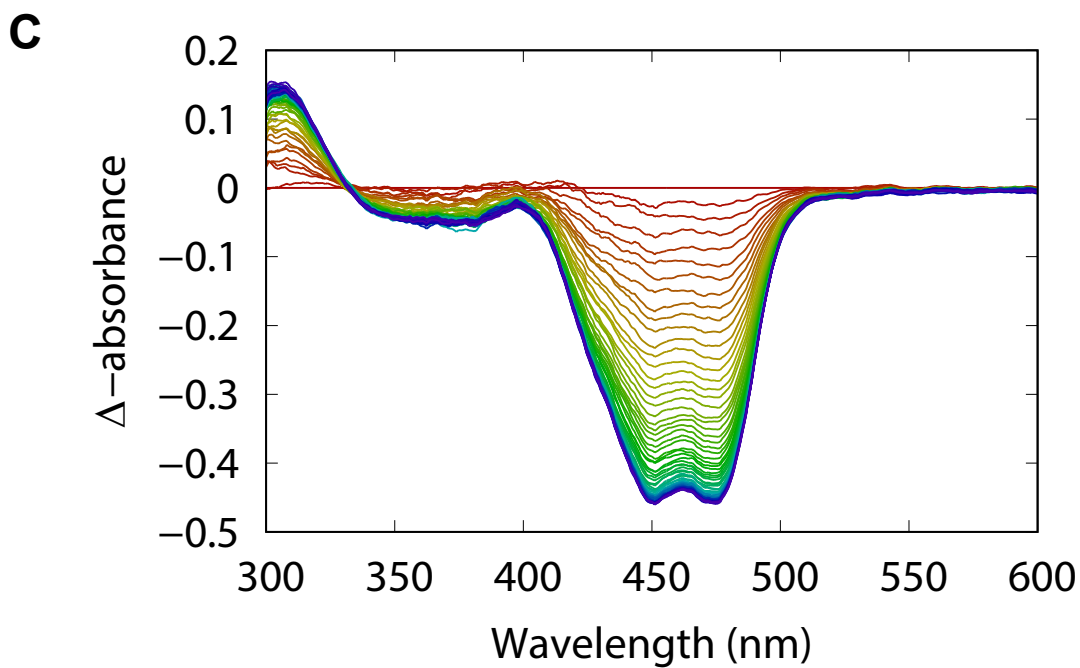

Supplement: FIG S4 [file mBio.00264-21-sf004.pdf]

**A**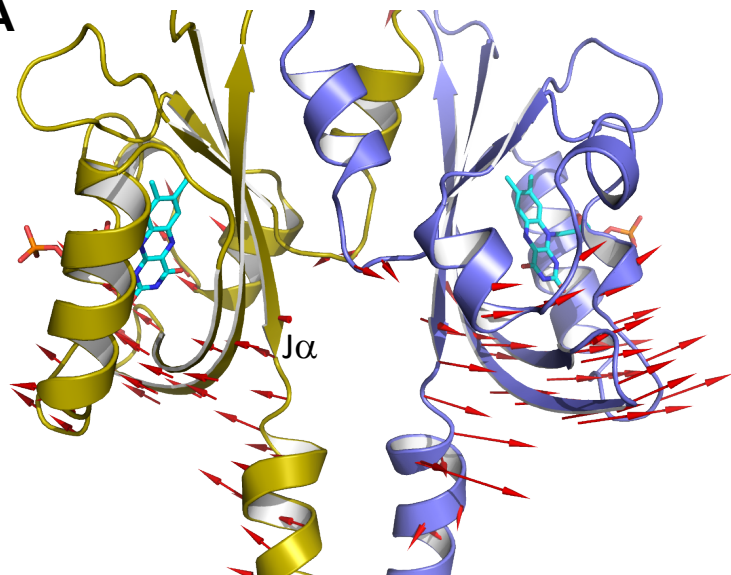**B**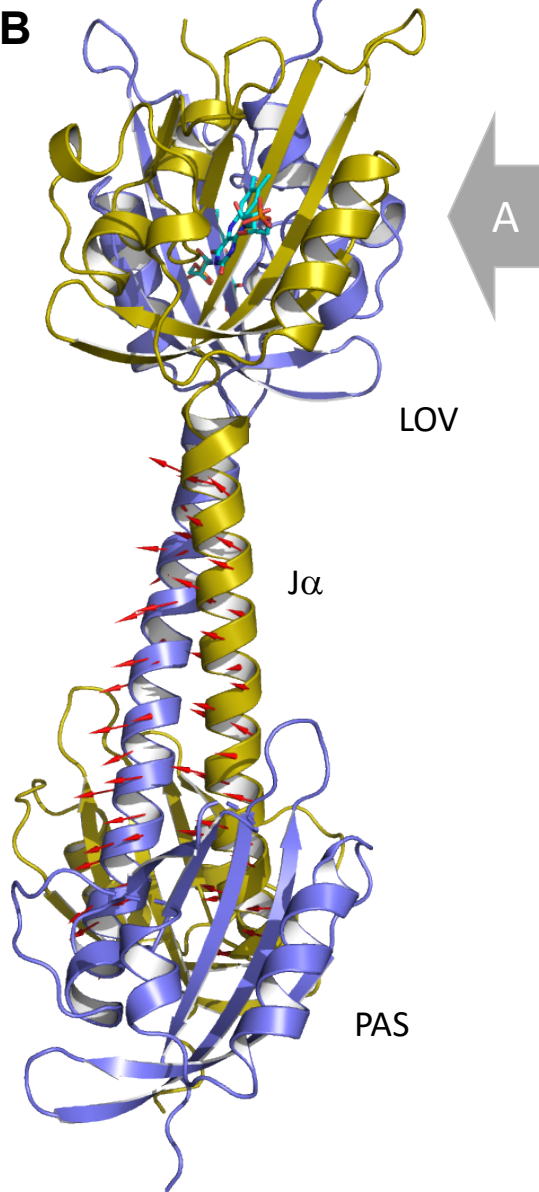**C**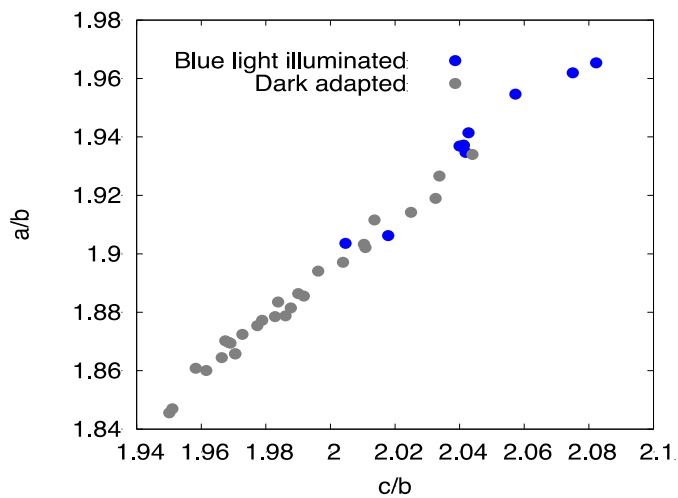

Supplement: FIG S6 [file mBio.00264-21-sf006.pdf]

**A**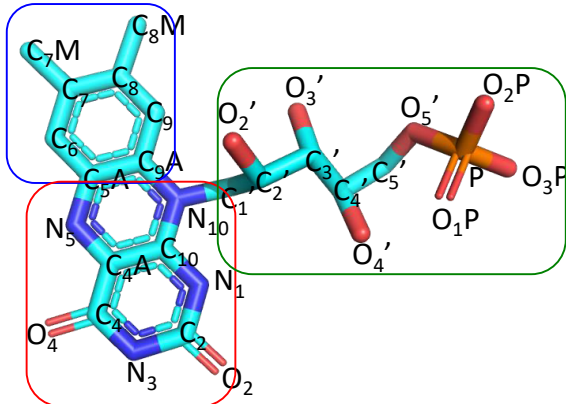**B**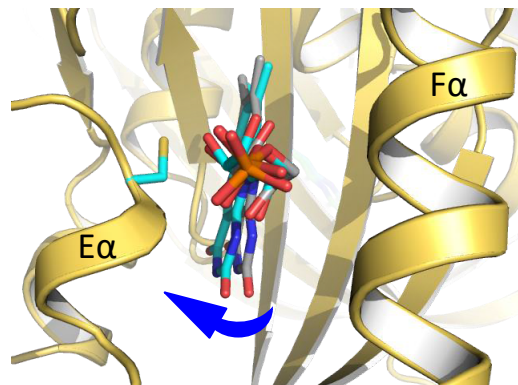**C**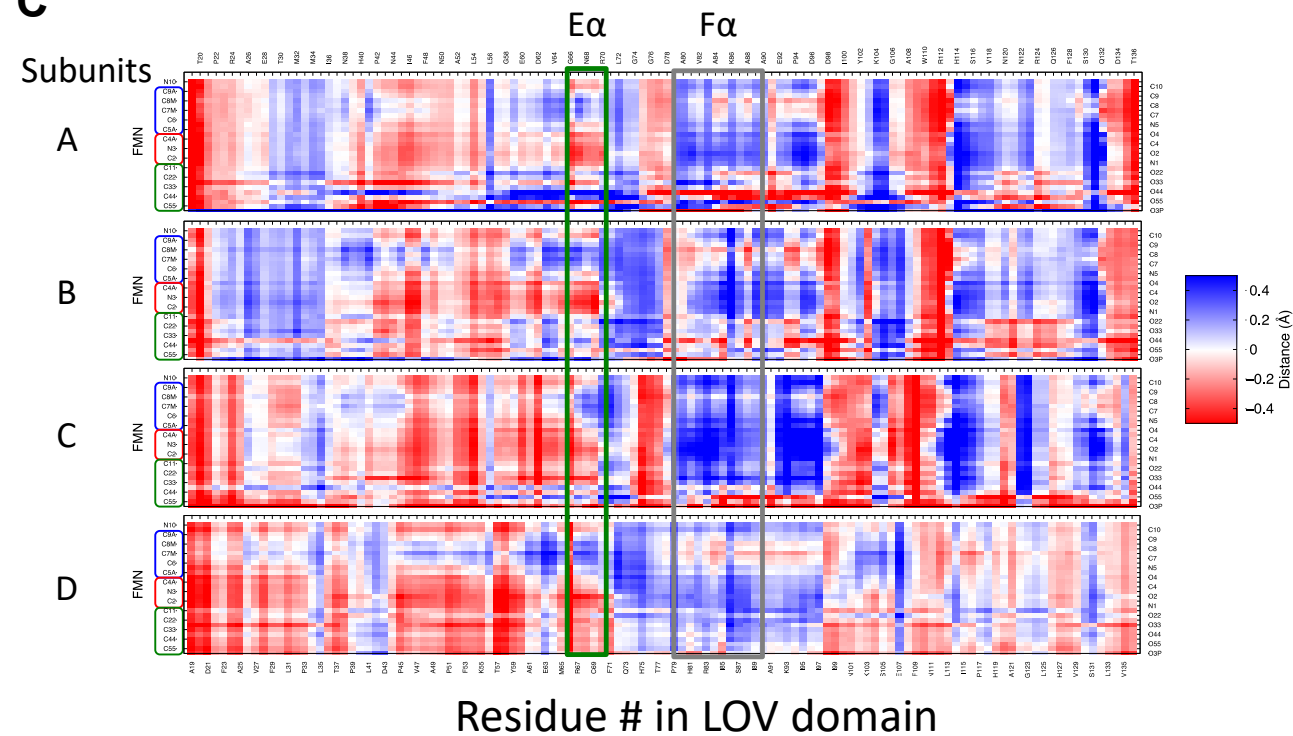

Supplement: FIG S7 [file mBio.00264-21-sf007.pdf]

**A**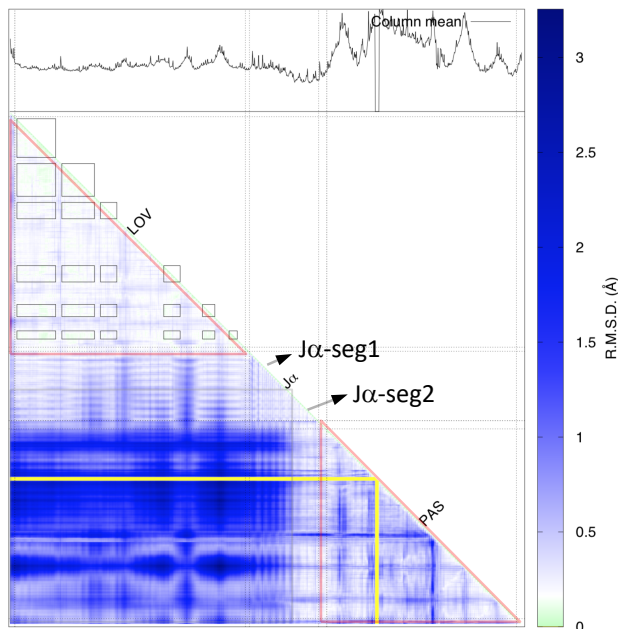**B**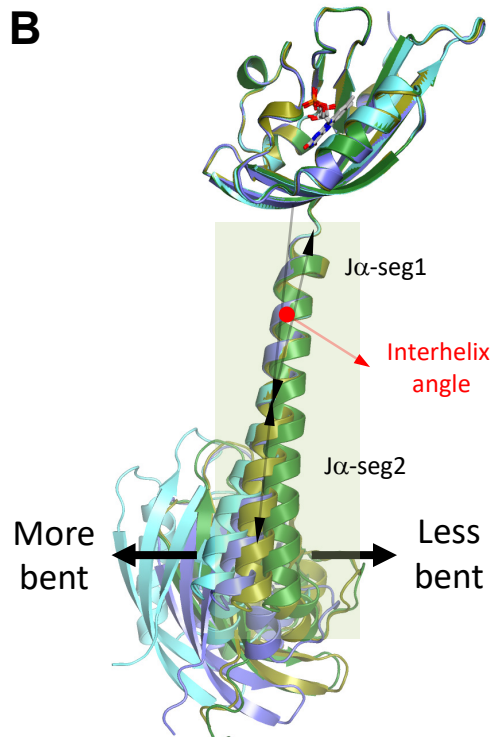**C**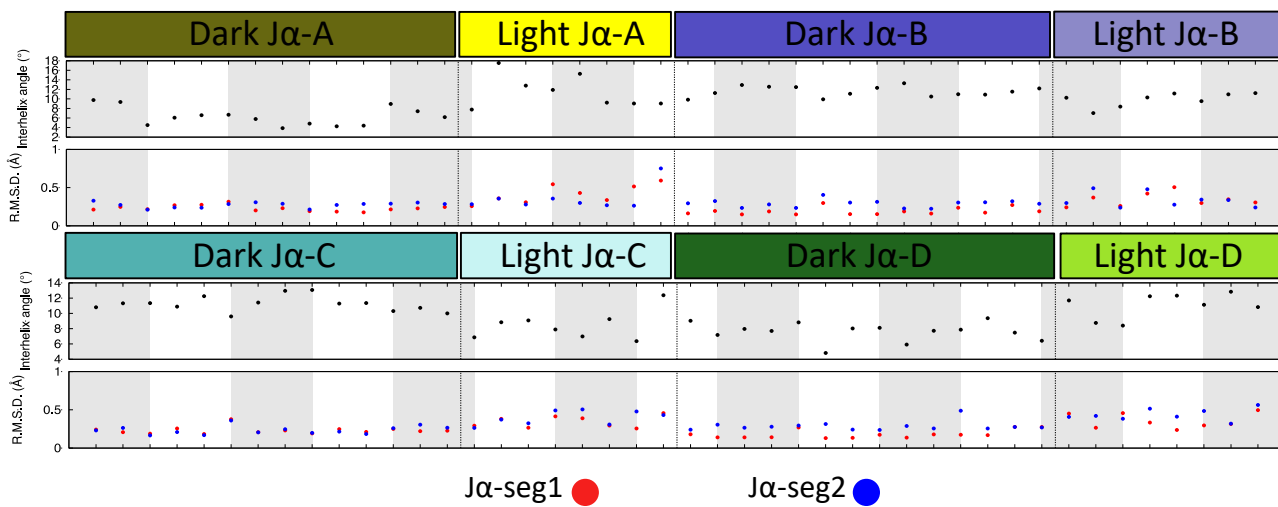

Supplement: FIG S8 [file mBio.00264-21-sf008.pdf]
