# Supplementary material for: Dimer Asymmetry and Light Activation Mechanism in Brucella Blue-Light Sensor Histidine Kinase
Source: mBio. 2021 Apr 20;12(2):e00264-21. doi: 10.1128/mBio.00264-21 (PMC8092228; doi:10.1128/mBio.00264-21)
Supplement: FIG S5 [file mBio.00264-21-sf005.pdf]

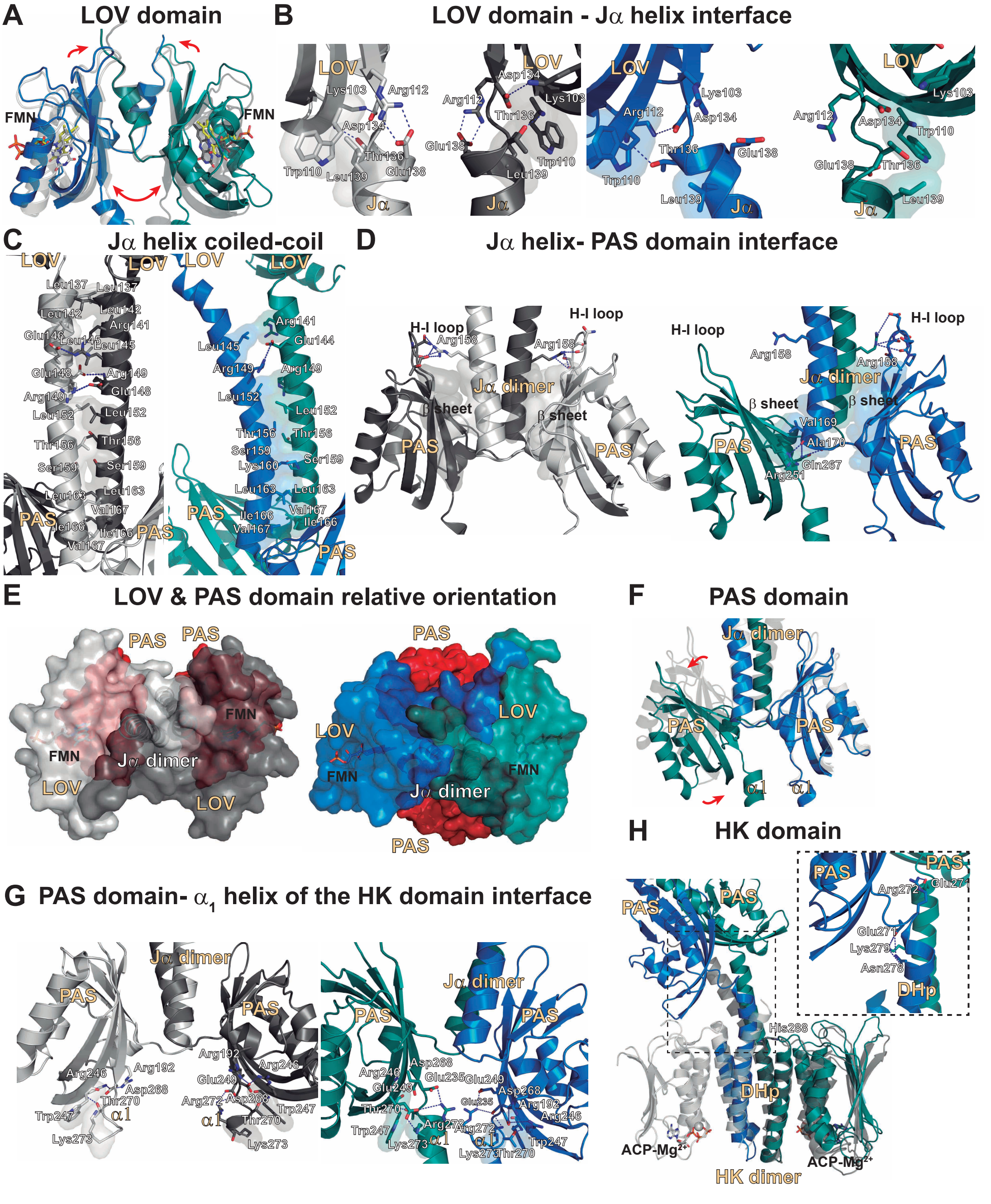
A
LOV domain
FMN
FMN
B
LOV domain - Jα helix interface
LOV
Lys103
Arg112
Asp134
Thr136
Glu138
Trp110
Leu139
Jα
C
Jα helix coiled-coil
LOV
Leu137
Leu142
Arg141
Glu146
Leu145
Glu148
Arg149
Glu144
Leu152
Thr156
Ser159
Lys160
Leu163
Val167
Ile166
PAS
D
Jα helix- PAS domain interface
H-I loop
Arg158
Jα dimer
β sheet
PAS
Val169
Ala170
Gln267
Arg251
E
LOV & PAS domain relative orientation
PAS
FMN
Jα dimer
LOV
F
PAS domain
Jα dimer
PAS
α1
G
PAS domain- α_1 helix of the HK domain interface
Jα dimer
PAS
Arg246
Arg192
Asp268
Thr270
Trp247
Lys273
Glu249
Arg272
Glu235
α1
H
HK domain
PAS
Arg272
Glu271
Lys279
Asn278
DHp
His288
ACP-Mg^{2+}
HK dimer
